# The Hybrid Histidine Kinase LadS Forms a Multicomponent Signal Transduction System with the GacS/GacA Two-Component System in *Pseudomonas aeruginosa*

**DOI:** 10.1371/journal.pgen.1006032

**Published:** 2016-05-13

**Authors:** Gaël Chambonnier, Lorène Roux, David Redelberger, Firas Fadel, Alain Filloux, Melissa Sivaneson, Sophie de Bentzmann, Christophe Bordi

**Affiliations:** 1 Laboratoire d’Ingénierie des Systèmes Macromoléculaires, Institut de Microbiologie de la Méditerranée, Aix-Marseille Université, CNRS UMR7255, Marseille, France; 2 Aix Marseille Université, CNRS, AFMB UMR 7257, 13288, Marseille, France; Max Planck Institute for Terrestrial Microbiology, GERMANY

## Abstract

In response to environmental changes, *Pseudomonas aeruginosa* is able to switch from a planktonic (free swimming) to a sessile (biofilm) lifestyle. The two-component system (TCS) GacS/GacA activates the production of two small non-coding RNAs, RsmY and RsmZ, but four histidine kinases (HKs), RetS, GacS, LadS and PA1611, are instrumental in this process. RetS hybrid HK blocks GacS unorthodox HK autophosphorylation through the formation of a heterodimer. PA1611 hybrid HK, which is structurally related to GacS, interacts with RetS in *P*. *aeruginosa* in a very similar manner to GacS. LadS hybrid HK phenotypically antagonizes the function of RetS by a mechanism that has never been investigated. The four sensors are found in most *Pseudomonas* species but their characteristics and mode of signaling may differ from one species to another. Here, we demonstrated in *P*. *aeruginosa* that LadS controls both *rsmY* and *rsmZ* gene expression and that this regulation occurs through the GacS/GacA TCS. We additionally evidenced that in contrast to RetS, LadS signals through GacS/GacA without forming heterodimers, either with GacS or with RetS. Instead, we demonstrated that LadS is involved in a genuine phosphorelay, which requires both transmitter and receiver LadS domains. LadS signaling ultimately requires the alternative histidine-phosphotransfer domain of GacS, which is here used as an Hpt relay by the hybrid kinase. LadS HK thus forms, with the GacS/GacA TCS, a multicomponent signal transduction system with an original phosphorelay cascade, *i*.*e*. H1_LadS_→D1_LadS_→H2_GacS_→D2_GacA_. This highlights an original strategy in which a unique output, i.e. the modulation of sRNA levels, is controlled by a complex multi-sensing network to fine-tune an adapted biofilm and virulence response.

## Author Summary

*P*. *aeruginosa* is able to switch from a planktonic to a sessile lifestyle by regulating the two small RNAs RsmY and RsmZ. The GacS/GacA TCS is the main system involved in their regulation; however, three HKs, RetS, LadS and PA1611, modulate this TCS. Here we elucidate the relationship between the LadS signaling pathway and the GacS/GacA TCS. We indeed show that LadS modulates GacS/GacA through an original phosphorelay mechanism where LadS utilizes its H1 and D1 domains and the H2 domain of the unorthodox GacS HK, thereby forming with GacS HK, a multicomponent signal transduction system. This multicomponent signal transduction system, first evidenced in this work, probably functions in a similar manner in some other pseudomonas species like *P*. *fluorescens* but not in all pseumonad genera as illustrated by *P*. *syringae*. Multicomponent signaling is an original and pertinent strategy for bacteria, in which a complex multi-sensing network controls a similar output (*i*.*e*. the modulation of levels of central regulatory RsmY and RsmZ sRNAs). This mechanism provides *P*. *aeruginosa* with the possibility of integrating at least two different signals, one from GacS and the other from LadS, to engage *P*. *aeruginosa* in a sessile lifestyle.

## Introduction

The ability of bacteria to survive in specific habitats requires coordination of appropriate gene expression in response to encountered environmental changes. It is interesting to note that the complexity of bacterial regulatory networks and the number of regulatory genes of bacterial genomes proportionally increase with the diversity of environments a bacterial species is able to survive [1]. In order to cope with the various environments they encounter, bacteria have evolved several sensing systems, including two-component systems (TCS) that monitor external and internal stimuli (nutrients, ions, temperature, redox states …), and translate these signals into adequate adaptive responses (for a review see, [1]).

A TCS comprises a histidine kinase (HK) protein or sensor mostly inserted into the inner membrane and a cognate partner known as the response regulator (RR). These two proteins function in their simplest version in a two-step phosphorelay mechanism, forming a classical TCS as follows: The detection of the stimulus by the periplasmic or cytoplasmic detection domain of the HK protein triggers autophosphorylation on a conserved histidine (H) residue of the transmitter domain H1. The phosphoryl group is then transferred on a conserved aspartate (D) residue present in the receiver or D domain of the cognate RR [2,3]. In some cases, the phosphorelay mechanism between the HK and the RR requires a four-step phosphorelay. In this case, the HK requires additional domains such as a receiver domain (D1). Although this D1 module could be on a separate protein, it is mostly fused to the HK. Then, an alternative histidine-phosphotransfer domain (Hpt or H2) can either be fused to the HK (H2) or form a third independent component in the cytoplasm called Hpt. The HK carrying both additional D1 and H2 domains are called unorthodox sensors while those carrying only the D1 domain are called hybrid sensors. Autophosphorylation of the first H residue of the H1 domain of hybrid or unorthodox HK initiates a phosphorelay such as H_H1_→D_D1_→H_H2 or Hpt_→D_RR_. In a few cases, the phosphorelay between the HK and the RR can be more complex and involves another TCS to form a multicomponent signal transduction system. The CblSTR signal transduction pathway of *Bukholderia cenocepacia*, which controls the expression of the cable pili, is such a system [4]. The *cblS* and *cblT* genes encode a hybrid and an unorthodox HK respectively, while the *cblR* gene encodes the cognate RR. While the first two steps of the phosphorelay require the H1 and D1 domains of CblS, the transphosphorylation of the D domain of CblR by CblS requires the H2 domain of CblT, which serves as a bridge component, increasing the complexity of the transduction pathway.

**Fig 1:**
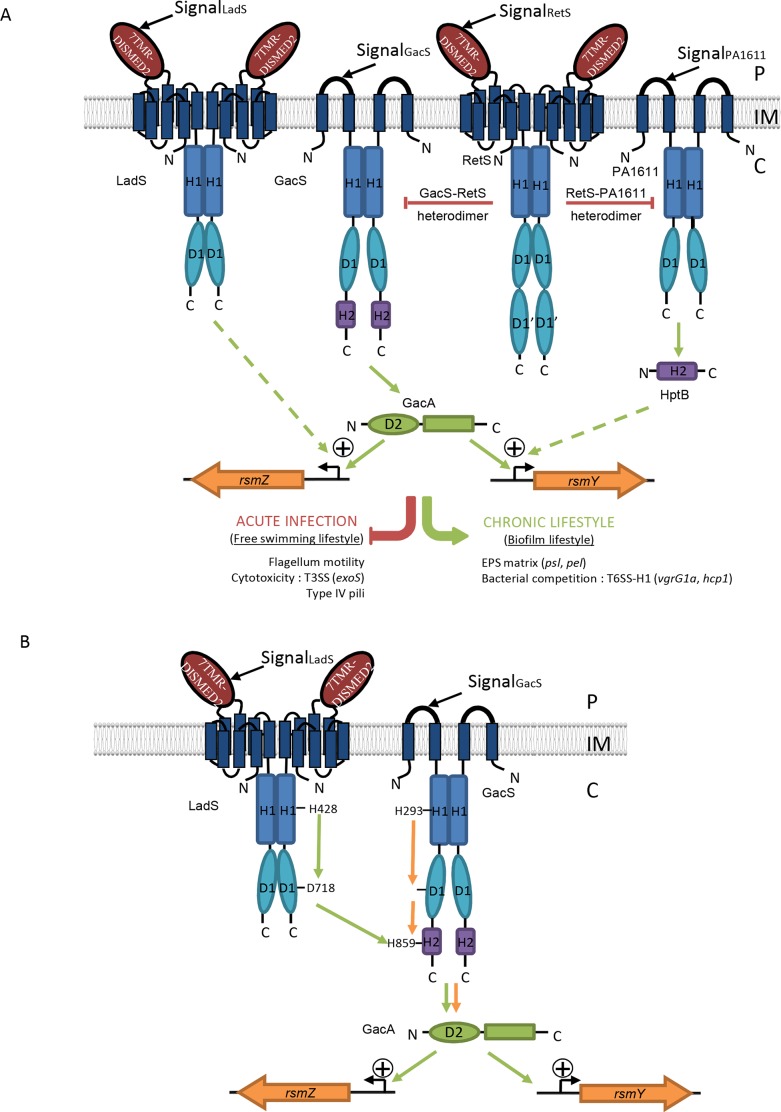
The GacSA-RetS-PA1611-LadS signaling network. (A) Current model for the regulatory elements influencing the expression of two sRNAs, RsmY and RsmZ. See text for details. (B) The multicomponent signal transduction system made of the LadS hybrid HK and the GacS/GacA TCS. In the presented model sustained by results obtained in the present study, this multicomponent signal transduction system made of the LadS hybrid HK and the GacS/GacA TCS forms a multiple-input system probably reflecting the variability of environmental conditions *P*. *aeruginosa* is faced with and may result in a range of gradations of chronic infection. IM (Inner Membrane), P (Periplasm), C (Cytoplasm).

*Pseudomonas aeruginosa* is a major human pathogen causing severe infections in vulnerable patients such as those with cystic fibrosis or hospitalized with cancer, burns and in intensive care units. It has become a major cause of nosocomial infections. Like other species, *P*. *aeruginosa* is able to switch from a planktonic (free swimming) to a sessile (biofilm) lifestyle and several TCSs play a key role in this switch [5–7]. Free swimming cells are characterized by an effective production and injection into host cells of effectors of the Type III secretion system (T3SS). In this free swimming lifestyle, they are thought to represent the vast majority of individuals causing acute infections [8] such as in sepsis, ventilator-associated pneumonia, and infections in postoperative wound and burn patients. In contrast, sessile cells are embedded in a biofilm community sealed by a matrix of exopolysaccharides (EPS) and DNA. In this state, the bacteria concomitantly secrete toxins delivered by the H1-Type VI secretion system (H1-T6SS), which are used for killing and competing with other species in this crowded and enclosed community [8–11]. Cells in biofilms are thought to be in conditions similar to those in chronic infections [12] such as in chronic obstructive pulmonary disease or cystic fibrosis. Several studies have reported an opposing regulation between the expression of molecular determinants involved in acute infection and those involved in chronic infection. Several TCSs have been described as key players controlling this transition, including the central and critical GacS/GacA TCS [13–17] (Fig 1A). GasS is an unorthodox HK with H1/D1/H2 domains. GacA is an RR functioning as a transcriptional regulator, which positively and exclusively controls the expression of two unique target genes encoding two small noncoding RNAs, RsmY and RsmZ [18]. Thus, RsmY and RsmZ have been proposed as key players in controlling the switch between planktonic and biofilm lifestyles [18,19]. These two sRNAs sequester the RNA-binding translational repressor RsmA and thus relieve RsmA binding from its target mRNAs. While bound to target sequences at the site of translational initiation, RsmA exerts a direct translational repression on a limited number of genes grouped in six operons [20], among which are genes encoding the H1-T6SS. Additionally, RsmA has been described as indirectly and positively controlling the expression of a substantial number of genes, including those encoding the T3SS participating in acute infection [20,21]. High expression of *rsmY* and *rsmZ* leads to massive biofilm formation due to the production of Pel EPS, and is coupled with H1-T6SS induction and T3SS repression (Fig 1A) [22]. In a *gacS* mutant, the absence of expression of these sRNAs results in an impaired biofilm formation and induction of T3SS expression [22]. In *P*. *aeruginosa*, expression of these two sRNAs is controlled by a complex and sophisticated regulatory network involving the GacS/GacA TCS but also other TCS pathways. The RetS hybrid HK represses expression of both *rsmY* and *rsmZ* genes by interfering with the GacS/GacA TCS activity [23]. The PA1611 hybrid HK induces expression of both *rsm* genes by counteracting the interfering effect of RetS on GacS [24]. The HptB regulatory pathway, which also intersects with the GacS/GacA TCS, only induces *rsmY* gene expression [22]. The LadS hybrid HK carrying H1/D1 domains has been shown to activate expression of the *rsmZ* gene. However, it controls target genes in a reciprocal manner as compared to RetS [25], suggesting that it may also control *rsmY* gene expression although this has not been demonstrated.

While the GacS/GacA TCS is widely distributed throughout the bacterial kingdom, the molecular switch formed by the hybrid LadS, PA1611 and RetS HKs is unique to the *Pseudomonas* species, though it can function in very different ways in phylogenetically related *Pseudomonas* species [26–28]. In *Pseudomonas fluorescens*, it has been proposed that LadS controls *rsmX*, *rsmY* and *rsmZ* expression through GacS, based on the observation that *gacS* or *gacA* mutations are epistasic to *ladS* mutation [29]. In *P*. *syringae*, the LadS, GacS and RetS HKs do not control the same targets and this is exemplified for T3SS whose LadS- and RetS-dependent control is GacS-independent in this bacterial species [26,27]. The absence of the D1 domain in *P*. *syringae* LadS HK may account for this GacS-independent T3SS regulation [27].

In this network (Fig 1A), the RetS regulatory pathway requires the presence of the GacS/GacA TCS to control *rsm* gene expression and this occurs via heterodimer formation between RetS HK and GacS HK [23,24,30], impeding GacS autophosphorylation and thereby preventing phosphorylation of its cognate RR GacA [23]. In *P*. *aeruginosa*, the PA1611 hybrid HK structurally related to GacS also interacts with RetS in *P*. *aeruginosa* in a very similar manner to GacS and RetS and its action is independent of LadS HK [24]. Furthermore, this interaction does not require the conserved phosphorelay residues of PA1611 [24]. Overall, it is still unclear whether in *P*. *aeruginosa*, LadS HK triggers *rsmZ* and possibly *rsmY* gene expression through GacS or another unorthodox HK or an Hpt protein connected to another TCS [25].

In the present study, we therefore addressed the questions of whether the *P*. *aeruginosa* LadS hybrid HK intersects with the GacS/GacA pathway and at which level by using combined genetic, biochemical and phenotypic approaches. We demonstrated that the LadS HK not only controls the expression of *rsmZ* but also the expression of *rsmY*, and thus modulates the production of Pel EPS, H1-T6SS and T3SS targets. We further demonstrated that LadS influences its target genes through the GacS/GacA TCS. Specifically, LadS autophosphorylates on its H1 domain, transfers the phosphoryl group on its D1 domain, and then subsequently to the H2 domain of GacS (Fig 1B). These results clearly showed that the LadS HK and the GacS/GacA TCS form a multicomponent signal transduction system, functioning in a mechanism clearly distinct from the one proposed for the RetS or the PA1611 hybrid HKs, the other members of the network.

## Results

### LadS HK signaling converges on RsmY or RsmZ to control its targets

In *P*. *aeruginosa*, the LadS HK had been shown to trigger *rsmZ* gene expression [25] but no data were available for its action on *rsmY* gene expression. We thus first addressed the question of whether LadS is able to control *rsmY* gene expression.

First, to override variable levels of *ladS* expression and activation (phosphorylation state) due to LadS signal, overexpression of *ladS* was first undertaken. This approach mimics a constitutive activation of HKs thereby enabling the study of the corresponding signaling pathway. Using chromosomal *rsmY–lacZ* and *rsmZ–lacZ* transcriptional fusions in the PAK genetic background (PAK*attB*::*rsmY-lacZ* and PAK*attB*::*rsmZ-lacZ*), we overexpressed the full-length *ladS* HK gene using the pBBR*ladS* plasmid [25]. Overexpression of *ladS* resulted in a significant increase in activity of both *rsm* fusions (S1A Fig). Maximal *rsm* gene expression was reached for both fusions at an OD_600nm_ of around 3.8 with an 82-fold and a 42-fold increase for *rsmY* and *rsmZ*, respectively, upon *ladS* overexpression. These results were confirmed by RT-qPCR (S1B Fig).

Because overexpression of genes encoding HKs can have adverse effects [31], we further assessed by RT-qPCR whether LadS produced at the chromosomal level could have the same impact on *rsmY* and *rsmZ* expression in the PAK and its isogenic mutant PAKΔ*ladS* strains. Levels of *rsmY* and *rsmZ* expression were, respectively, reduced by a 17-fold and an 11-fold factor in the *ladS* mutant as compared to the wild-type strain (S1B Fig). This suggests that the *ladS* gene is indeed expressed and that LadS signal, although unknown, is present in our testing conditions. This further proved that LadS controls *rsmY* and *rsmZ* gene expression at the basal level. The basal level of *ladS* expression in the wild-type strain PAK was further determined by RT-qPCR. An absolute number of 4,600 copies of *ladS* gene mRNA copies per μg of total RNA retrotranscribed was monitored in the wild-type PAK strain bearing or not the mild copy empty vector pBBRMCS4 and no copy was detectable in its counterpart *ladS* mutant. This number increased to 130,000 copies when *ladS* gene was expressed from the pBBRMCS4 vector (S1C Fig). From these results, overexpression of *ladS* with a mild copy vector appeared to be a suitable way to investigate the LadS signaling pathway: it can reproduce LadS signaling without adverse effect since the increase in *rsm* gene expression observed in *ladS* overexpression conditions was consistent with the one obtained under *ladS* basal expression (S1A and S1B Fig).

**Fig 2:**
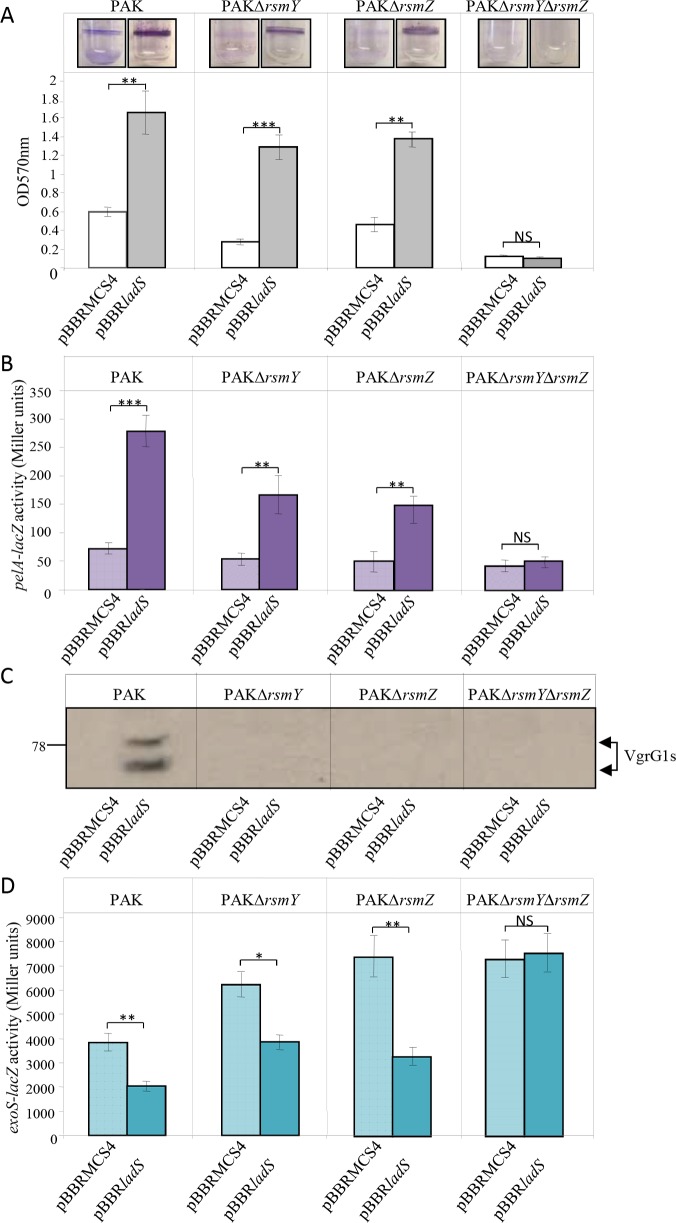
Biofilm production, Pel EPS expression, H1-T6SS production and T3SS expression in the LadS signaling pathway. The pBBR*ladS* plasmid containing the *ladS* HK gene (dark bars) and the pBBRMCS4 corresponding empty cloning vector (light bars) were conjugated in the PAK, PAKΔrsmY, PAKΔ*rsmZ* or PAKΔ*rsmY*Δ*rsmZ* strains. (A) Biofilm production in glass tubes was illustrated (upper panel) and quantified after Crystal Violet-staining (lower panel). Corresponding levels of biofilm production represent mean values and standard deviations obtained from three independent experiments. (B) Activity of the *pelA–lacZ* transcriptional chromosomal fusion was monitored in the same strains with the pBBR*ladS* plasmid containing the *ladS* HK gene (dark violet bars) and the pBBRMCS4 corresponding empty cloning vector (light violet bars) after 4 hours of growth (OD_600nm_≈3.5). Corresponding β-galactosidase activities are expressed in Miller units and correspond to mean values (with error bars) obtained from three independent experiments. (C) Production of the H1-T6SS VgrG1s proteins was detected in whole cell extracts using western blot with an anti-VgrG1 polyclonal antibody. Numbers on the left side correspond to molecular weight standards (kDa). (D) Activity of the *exoS–lacZ* transcriptional chromosomal fusion was monitored in the same strains with the pBBR*ladS* plasmid containing the *ladS* HK gene (dark royal blue bars) and the pBBRMCS4 corresponding empty cloning vector (light royal blue bars) after 6 hours of growth (OD_600nm_≈4). Corresponding β-galactosidase activities are expressed in Miller units and correspond to mean values (with error bars) obtained from three independent experiments. Wilcoxon-Mann-Whitney tests were performed and *, **, *** and ns referred to p<0.05, p<0.01 and p<0.001 and nonsignificant difference, respectively.

LadS was shown to antagonistically control several of the RetS targets such as Pel, T6SS and T3SS [25]. We next investigated whether it exclusively occurs through the two sRNAs RsmY and RsmZ. The pBBR*ladS* and pBBRMCS4 plasmids were separately conjugated in the PAK, PAKΔ*rsmY*, PAKΔ*rsmZ* and PAKΔ*rsmYΔrsmZ* strains. Overexpression of the *ladS* HK gene resulted in a 2.65-, 4.3- and 2.8-fold increase in biofilm formation in PAK and in the single *rsmY* and *rsmZ* mutants, respectively, while the double mutation abrogated biofilm formation (Fig 2A). Since in the PAK background the biofilm built-in response to the activation of the *rsm* genes is mostly dependent on *pel* gene expression [10,22], we further investigated whether the *ladS*-dependent biofilm formation could rely on the transcriptional activity of the *pel* locus. The activity of the chromosomal *pelA–lacZ* transcriptional fusion was therefore assessed in the PAK, PAKΔ*rsmY*, PAKΔ*rsmZ* and PAKΔ*rsmYΔrsmZ* strains transformed with either the pBBR*ladS* or the pBBRMCS4 plasmids. Upon *ladS* overexpression, the β-galactosidase activity of the *pelA* transcriptional fusion measured after 4 hours of growth (OD_600nm_≈3.5) was significantly induced in the wild-type strain, intermediately induced in the *rsmY* or *rsmZ* single mutants and abolished in the double *rsmYrsmZ* mutant (Fig 2B). Another known LadS target is the H1-T6SS, whose production was further checked by immunodetection of the VgrG1 proteins. While *ladS* HK overexpression induced production of VgrG1 proteins in the PAK strain, these T6SS proteins were undetectable in the PAKΔ*rsmY*, PAKΔ*rsmZ* and PAKΔ*rsmYΔrsmZ* strains (Fig 2C). Finally, LadS control on T3SS was checked using a chromosomal *exoS–lacZ* transcriptional fusion in the PAK, PAKΔ*rsmY*, PAKΔ*rsmZ* and PAKΔ*rsmYΔrsmZ* strains, which had received the pBBR*ladS* plasmid or the corresponding empty vector pBBRMCS4. Cells were grown in the presence of EDTA, a Ca^2+^ chelator, a condition known to activate T3SS expression. Upon *ladS* overexpression, the β-galactosidase activity of the *exoS* fusion was reduced 2-fold in the wild-type strain, 1.65-fold in the *rsmY* mutant and 1.9-fold in the *rsmZ* mutant but had no effect in the double *rsmYrsmZ* mutant (Fig 2D). These *ladS*-overexpression effects on *pelA*, *vgrG1b* and *exoS* genes were confirmed by RT-qPCR (S1B Fig). The LadS signaling pathway was found to exert the same control on these targets (Pel, T6SS and ExoS) for *ladS* expression at the basal chromosomal level (S1B Fig).

Taken together, these results demonstrated that like RetS, LadS HK controls the expression of both *rsmY* and *rsmZ* genes and impacts biofilm formation and Pel production, H1-T6SS and T3SS. Since these sRNAs are the exclusive targets of the GacA RR, our results strongly suggest that such control could occur through the GacS/GacA pathway.

### LadS signaling pathway requires GacS and GacA

**Fig 3:**
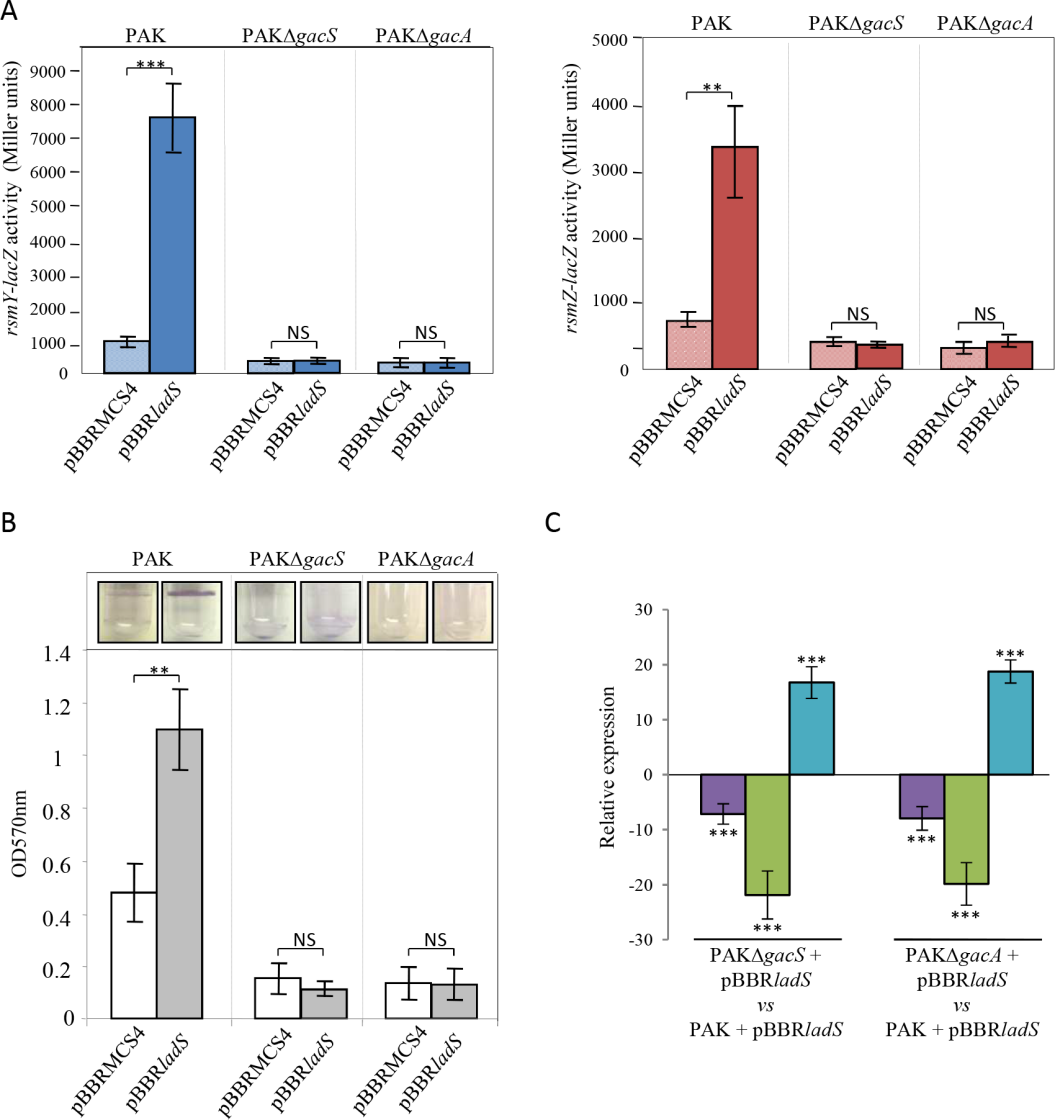
Role of the GacS/GacA TCS in the LadS signaling pathway. The pBBR*ladS* plasmid containing the *ladS* HK gene (dark bars) and the pBBRMCS4 corresponding empty cloning vector (light bars) were conjugated in the PAK, PAKΔgacS or PAKΔ*gacA* strains. (A) Activities of the *rsmY–lacZ* (left panel, blue bars) and *rsmZ–lacZ* (right panel, brick-red-colored bars) transcriptional chromosomal fusions were monitored after 6 hours of growth (OD_600nm_≈4) and corresponding β-galactosidase activities are expressed in Miller units and correspond to mean values (with error bars) obtained from three independent experiments. (B) Biofilm production in glass tubes was illustrated (upper panel) and quantified after crystal violet-staining (lower panel). Corresponding levels of biofilm production represent mean values and standard deviations obtained from three independent experiments. Wilcoxon-Mann-Whitney tests were performed and *, **, *** and ns referred to p<0.05, p<0.01 and p<0.001 and nonsignificant difference, respectively. (C) Transcript levels of PelA (violet bars), VgrG1b (T6SS) (green bars) and ExoS (T3SS) (royal blue bars) were monitored by RT-qPCR in PAK, PAKΔ*gacS* and PAKΔ*gacA* strains with the pBBR*ladS* plasmid containing the *ladS* HK gene and the pBBRMCS4 corresponding empty cloning vector and fold induction was presented for the two mutant strains as compared to the PAK strain. Moderated t-tests were performed; *, ** and *** referred respectively to p<0.05, p<0.01 and p<0.001.

We next investigated whether in *P*. *aeruginosa*, LadS signaling converges on the GacS/GacA TCS, as previously demonstrated for RetS [16,23] and HptB [22] signaling pathways. We first examined whether *ladS* HK overexpression promotes *rsm* gene expression in *gacA* and *gacS* mutants by monitoring the activity of chromosomal *rsmY-lacZ* and *rsmZ-lacZ* fusions in both mutants. Overexpression of *ladS* was no longer able to induce *rsmY* or *rsmZ* expression in both mutants (Fig 3A), indicating that LadS control of *rsm* gene expression occurs through the GacS/GacA TCS. This was confirmed by examining phenotypes highly dependent on GacS/GacA and on the sRNAs RsmY and RsmZ. As illustrated in Fig 3B, the ability of LadS to promote biofilm in the wild-type strain was fully abolished in both *gacS* and *gacA* mutants. Similarly, LadS was unable to control the expression of *pel*, *vgrG1b* and *exoS* genes in *gac* mutants (Fig 3C). These results confirmed that in *P*. *aeruginosa*, LadS-dependent induction of *rsmY* and *rsmZ* gene expression and of their targets is strictly dependent on the GacS/GacA TCS.

### H1 domain of the LadS HK does not heterodimerize with H1 domains of GacS or RetS HK

It was shown earlier that RetS forms a heterodimer via its H1 domain, with the H1 domain of GacS, and that such heterodimerization prevents GacS autophosphorylation independently of any phosphorelay residue of RetS HK [23]. Because of the antagonism between RetS and LadS HK, we next examined whether LadS HK could form an active heterodimer with GacS HK or an inactive heterodimer with RetS HK counteracting the inhibitory effect of RetS HK on GacS HK by using pull-down and two-hybrid experiments.

**Fig 4:**
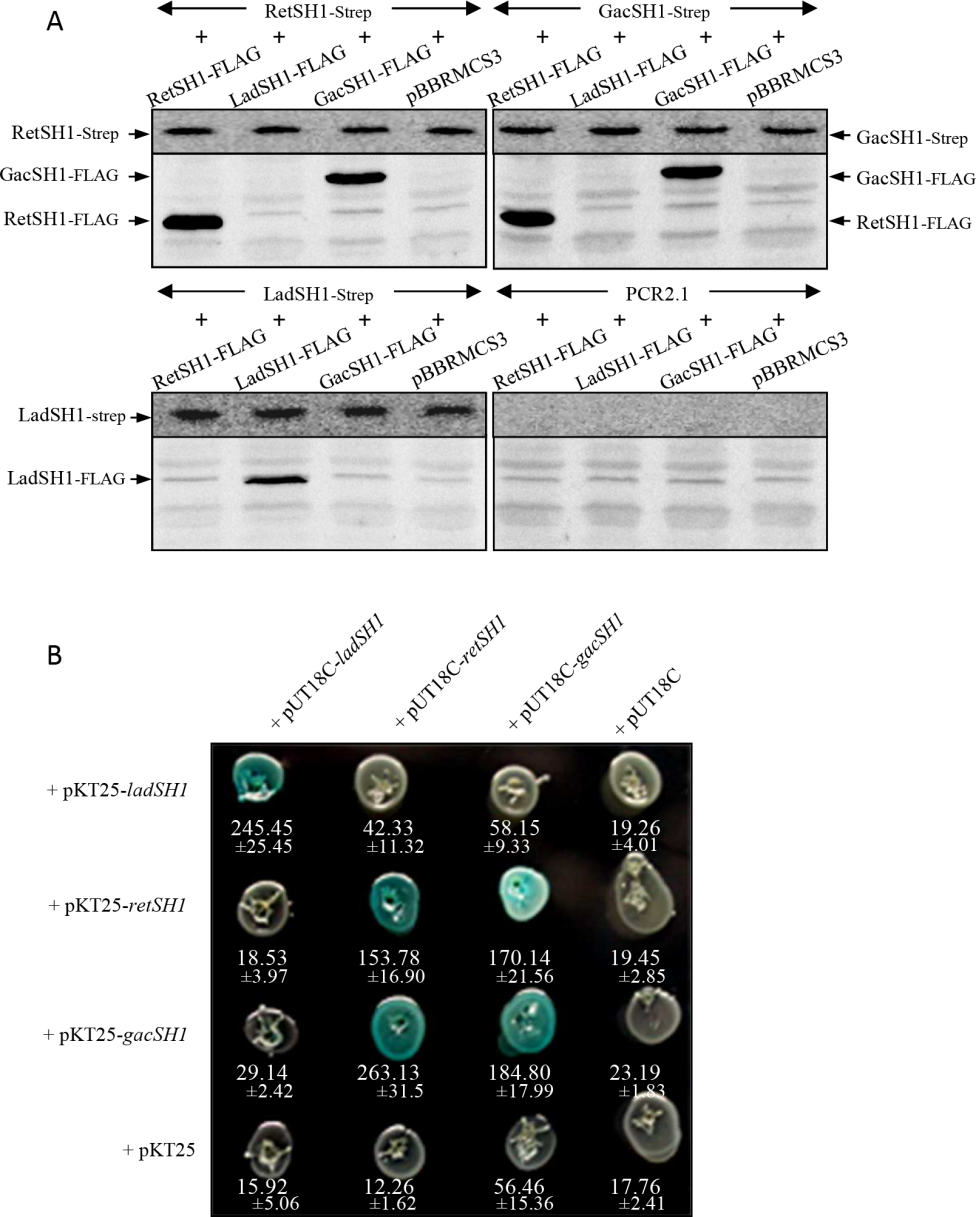
Interactions between H1 domains of the LadS hybrid HK, the GacS unorthodox HK and the RetS hybrid HK using pull-down and two-hybrid experiments. (A) Pull-down experiments. N-terminal FLAG or Strep versions of the H1 domain of GacS, LadS and RetS HKs were constructed in pBBRMCS3 and pCR2.1 vectors, respectively, and expressed in *E*. *coli*. Cell lysates were immunoprecipitated using anti-Strep antibody-coupled beads, and FLAG and Strep derivatives were further detected using StrepTactin Alkaline Phosphatase conjugate (upper panel) and anti-FLAG antibody detection (lower panel). (B) In two-hybrid experiments, the *ladSH1*, *retSH1* and *gacSH1* DNA regions were cloned into the two-hybrid pUT18C or pKT25 vectors and corresponding vectors were co-transformed in BTH101 cells that were further streaked on LB plates containing X-gal. A blue color of colonies reflects interaction between chimeric proteins, while white color attests to the absence of interaction. The interactions were further quantified by measuring the corresponding ß-galactosidase levels expressed in Miller units (values and standard deviations of 3 independent clones below corresponding colonies).

The different N-terminal-tagged (FLAG or Strep) versions of H1 domains of GacS, LadS and RetS HKs were efficiently co-produced in *E*. *coli* (S2 Fig). Pull-down experiments were performed using anti-Strep antibody-coupled beads. The LadSH1-FLAG protein was only pulled down in LadSH1-Strep-producing cells (Fig 4A, lower left panel) while the RetSH1-FLAG and GacSH1-FLAG proteins were pulled down in both RetSH1-Strep- and GacSH1-Strep-producing cells (Fig 4A, upper left and right panel), confirming the capacity of the H1 RetS and the H1 GacS domains to form homo- and heterodimers [23,24,32]. The absence of heterodimer formation involving the H1 domain of LadS HK was further confirmed by using two-hybrid experiments. As shown in Fig 4B, the H1 domain of LadS HK was unable to interact with either the H1 domain of GacS HK or the H1 domain of RetS HK. The H1 domain of each HK, LadS, RetS and GacS was able to homodimerize and interaction was also observed between H1 domains of RetS HK and GacS HK reflecting RetS/GacS heterodimerization as previously reported [23] (Fig 4B shows strains having received both vectors on X-gal-containing plates as well as corresponding levels of measured ß-galactosidase activities). Altogether, these results demonstrate that the H1 domain of LadS HK does not form heterodimers with H1 domains of RetS and of GacS, while H1 domains of RetS and GacS HKs form homo- and heterodimers.

### LadS signaling pathway requires functional H1 and D1 domains

**Fig 5:**
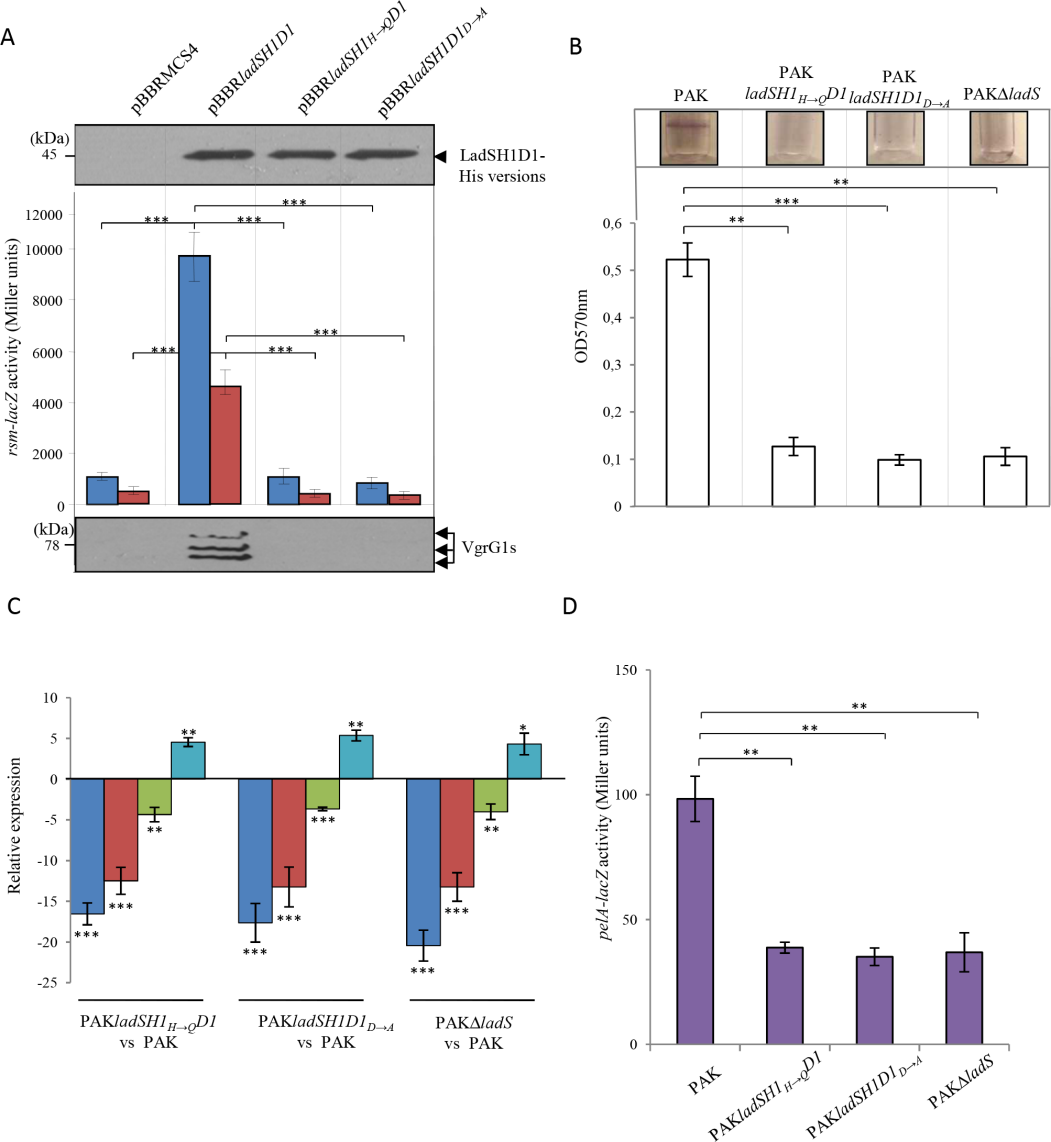
H1 and D1 domain involvement of the LadS hybrid HK in the LadS signaling pathway. (A) The pBBR*ladSH1D1* plasmid containing the *ladSH1D1* cytoplasmic DNA region of the LadS hybrid HK fused to a C-terminal His-tag, the pBBR*ladSH1*_*H→Q*_*D1* and pBBR*ladSH1D1*_*D→A*_ variant plasmids and the pBBRMCS4 corresponding empty cloning vector were conjugated in the PAK strain. Production of the corresponding cytoplasmic versions of LadS was checked in whole cell extracts using western blot and a monoclonal anti-His antibody. Numbers on the left side are molecular weight standards (kDa) (upper panel). Activity of the *rsmY–lacZ* (blue bars) and *rsmY–lacZ* (brick-red-colored bars) transcriptional chromosomal fusions were monitored after 6 hours of growth (OD_600nm_≈4) and corresponding β-galactosidase activities are expressed in Miller units and correspond to mean values (with error bars) obtained from three independent experiments. Wilcoxon-Mann-Whitney tests were performed and *, **, *** and ns referred to p<0.05, p<0.01 and p<0.001 and nonsignificant difference, respectively (middle panel). Production of the H1-T6SS VgrG1 protein was detected in whole cell extracts using western blot with an anti-VgrG1 polyclonal antibody. Numbers on the left side are molecular weight standards (kDa) (lower panel). (B) Biofilm production in glass tubes of PAK, PAKΔ*ladS* and of point chromosomal mutants PAK*ladSH1*_*H→Q*_*D1* and PAK*ladSH1D1*_*D→A*_ was presented (upper panel) and quantified after crystal violet-staining and extraction (lower panel). Corresponding levels of biofilm production represented by mean values and standard deviations were obtained from three independent experiments. Wilcoxon-Mann-Whitney tests were performed and *, **, *** and ns referred to p<0.05, p<0.01 and p<0.001 and nonsignificant difference, respectively. (C) Transcript levels of RsmY (blue bars), RsmZ (brick-red-colored bars), VgrG1b (T6SS) (green bars) and ExoS (T3SS) (royal blue bars) were monitored in PAK, PAKΔ*ladS* and in point chromosomal mutants PAK*ladSH1*_*H→Q*_*D1* and PAK*ladSH1D1*_*D→A*_ strains using RT-qPCR. Fold induction was presented for the three mutant strains as compared to the PAK strain. Moderated t-tests were performed; *, ** and *** referred respectively to p<0.05, p<0.01 and p<0.001. (D) Activity of the *pelA–lacZ* (violet bars) transcriptional chromosomal fusion was monitored after 6 hours of growth (OD_600nm_≈5) and corresponding β-galactosidase activities are expressed in Miller units and correspond to mean values (with error bars) obtained from three independent experiments. Statistical tests were performed and ** referred to p<0.01.

We next tested whether functional H1 and D1 domains of the LadS hybrid HK are required for the LadS-dependent signaling pathway. For that purpose, we engineered a truncated His-tagged version of the whole LadS cytoplasmic part of the HK including the H1 and D1 domains thus referred to as LadSH1D1 yielding to pBBR*ladSH1D1* (S3 Fig). We then performed site-directed mutagenesis in the corresponding wild-type LadS protein, to generate LadSH1_H→Q_D1 and LadSH1D1_D→A_ versions in which histidine residue in position 428 of the H1 domain and aspartate residue in position 718 of the D1 domain were substituted with a glutamine and an alanine, respectively yielding pBBR*ladSH1*_*H→Q*_*D1* and pBBR*ladSH1D1*_*D→A*_ (S3 Fig). All truncated versions, although lacking the transmembrane domains anchoring them in the inner membrane, were effectively and equivalently produced in the cytoplasm as detected by western blot (Fig 5A, upper panel). The LadSH1D1 version was able to promote expression of *rsmY* and *rsmZ* gene (Fig 5A, middle panel) to levels comparable with those obtained with the full-length version (compare *rsmZ-lacZ* activities with those presented in Fig 3A) while the LadSH1_H→Q_D1 and LadSH1D1_D→A_ versions were unable to do so (Fig 5A, middle panel). Moreover, the wild-type LadSH1D1 version was able to induce the production of VgrG1 proteins while the LadSH1_H→Q_D1 and LadSH1D1_D→A_ versions were not (Fig 5A, lower panel). To confirm these results, we directly engineered the same point mutations in the *ladS* gene of the PAK strain, leading to the PAK*ladSH1*_*H→Q*_*D1* and PAK*ladSH1D1*_*D→A*_ strains. Equivalent results were obtained with point chromosomal *ladS* mutants of the full-length *ladS* gene and *ladS* mutant for biofilm (Fig 5B), expression of *rsmY*, *rsmZ*, T6SS-H1 and T3SS genes (Fig 5C) and EPS production (Fig 5D). Taken together, these results demonstrated that the LadS signaling pathway requires both the histidine residue of its H1 domain and the aspartate residue of its D1 domain to activate *rsm* gene expression and further target genes.

### LadS requires only the H2 domain of GacS to control its target genes

**Fig 6:**
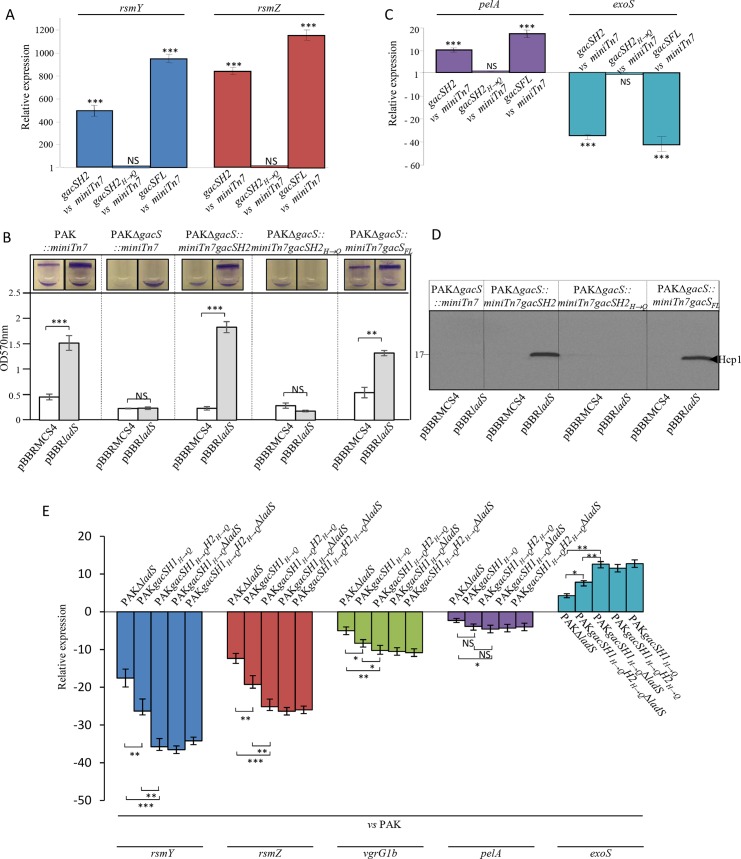
Involvement of the H2 domain of the GacS unorthodox HK in the LadS signaling pathway. The pBBR*ladS* plasmid containing the *ladS* HK gene and the pBBRMCS4 corresponding empty cloning vector were conjugated in the PAKΔ*gacS* strain in which the *gacSH2* or *gacSH2*_*H→Q*_ gene versions or the corresponding suicide vector were chromosomally integrated at the Tn7 site. (A) RsmY (blue bars) and RsmZ (brick-red-colored bars) transcript levels were monitored using RT-qPCR and fold induction was presented in the strains PAKΔ*gacS*::*miniTn7gacSH2* (*gacSH2*) and PAKΔ*gacS*::*miniTn7gacSH2*_*H→Q*_ (*gacSH2*_*H→Q*_) as compared to the PAKΔ*gacS*::*miniTn7* strain (*miniTn7*). (B) Biofilm production in glass tubes was illustrated (upper panel) and quantified after crystal violet-staining (lower panel). Corresponding levels of biofilm production represent mean values and standard deviations obtained from three independent experiments. Wilcoxon-Mann-Whitney tests were performed; ** and ns referred to p<0.01 and nonsignificant difference. **C.** PelA (violet bars) and ExoS (royal blue bars) transcript levels were monitored using RT-qPCR and fold induction was presented in the strains PAKΔ*gacS*::*miniTn7gacSH2* (*gacSH2*) and PAKΔ*gacS*::*miniTn7gacSH2H→Q* (*gacSH2H→Q*) as compared to the PAKΔ*gacS*::*miniTn7* strain (*miniTn7*). (D) Production of the H1-T6SS Hcp1 proteins was detected in whole cell extracts using western blot with an anti-Hcp1 polyclonal antibody. Numbers on the left side are molecular weight standards (kDa). Moderated t-tests were performed and *, **, *** and ns referred to p<0.05, p<0.01 and p<0.001 and nonsignificant difference, respectively. (E) Transcript levels of RsmY (blue bars), RsmZ (brick-red-colored bars), VgrG1 (green bars), PelA (violet bars) and ExoS (royal blue bars) were monitored using RT-qPCR. Fold induction was presented in the strains PAK, PAKΔ*ladS*, PAK*gacSH1*_*H→Q*_, PAK*gacSH1*_*H→Q*_Δ*ladS*, PAK*gacSH1*_*H*_→_*Q*_*H2*_*H*_→_*Q*_ and PAK*gacSH1*_*H*_→_*Q*_*H2*_*H*_→_*Q*_Δ*ladS* in order to disable autophosphorylation of the GacSH1 domain and the functionality of the GacsH2 domain, respectively. Moderated t-tests were performed and *, **, *** and ns referred to p<0.05, p<0.01 and p<0.001 and nonsignificant difference, respectively.

Since we evidenced that LadS requires its phosphorelay residues for signaling, we next investigated whether such phosphorelay requires the H2 domain of GacS. To test this, we engineered a truncated version of the GacS HK formed from its H2 domain (GacSH2) and its counterpart version GacSH2_H→Q_ in which the histidine residue in position 859 was mutated into a glutamine residue (S3 Fig), the corresponding H863 residue being crucial for GacS H2 domain activity in *P*. *fluorescens* CHA0 [33]. The corresponding *gacSH2* and *gacSH2*_*H→Q*_ gene versions as well as the *gacS* full-length gene (*gacS*_*FL*_) were introduced into the chromosome of a *gacS* mutant at the miniTn7 site in which we further introduced the pBBR*ladS* or the corresponding empty vector. GacSH2 was able to trigger a ≈ 600- and a 800-fold induction of *rsmY* and *rsmZ* transcript levels in cells overexpressing *ladS* as compared to cells carrying the corresponding empty vector, respectively. In contrast, GacSH2_H→Q_ could not transduce LadS signaling (Fig 6A). Upon *ladS* overexpression, the production of GacSH2 was sufficient to induce biofilm formation (Fig 6B), *pel* expression assessed by RT-qPCR (Fig 6C), T3SS gene repression (Fig 6C) and production of T6SS proteins (Fig 6D) while the GacSH2_H→Q_ had no ability to affect the *rsm*-dependent phenotypes tested here. Interestingly, when the *gacS*_*FL*_ gene was introduced in the *gacS* mutant, the complementation level of each *rsm*-dependent phenotype under *ladS* overexpression was similar to that obtained with the *gacSH2* gene version. As these results were obtained upon LadS overproduction, we further addressed the question of whether the LadS phosphotransfer to GacS occurs at the natural levels of expression of LadS and GacS HKs. We thus engineered the mutations *gacSH1*_*H→Q*_*H2* and *gacSH1*_*H*_→_*Q*_*H2*_*H*_→_*Q*_ in the wild-type and *ladS* mutant strains to disable autophosphorylation of the GacSH1 domain and the functionality of the GacsH2 domain, respectively. These strains were evaluated for their capacity to control the expression of *rsmY*, *rsmZ*, T6SS, *pelA* and T3SS genes by RT-qPCR (Fig 6E). GacSH1 domain autophosphorylation disabled in the strain PAK*gacSH1*_*H→Q*_ led to a reduction of *rsmY*, *rsmZ*, *pelA* and T6SS gene expression and an induction of T3SS gene expression as compared to the PAK strain. In this genetic context, further alteration of GacSH2 domain functionality (PAK*gacSH1*_*H→Q*_*H2*_*H→Q*,_ strain) resulted in a higher impact on *rsmY*, *rsmZ*, T6SS, *pelA* and T3SS gene expression. We further engineered deletion of the *ladS* gene in these two strains yielding respectively the PAK*gacSH1*_*H→Q*_Δ*ladS* and PAK*gacSH1*_*H→Q*_*H2*_*H→Q*_Δ*ladS* strains. The *ladS* gene deletion in the PAK*gacSH1*_*H→Q*_ strain impacted *rsmY*, *rsmZ*, T6SS, *pelA* and T3SS gene expression to a similar level as observed in the PAK*gacSH1*_*H→Q*_*H2*_*H→Q*_ strain and *ladS* gene deletion in the PAK*gacSH1*_*H→Q*_*H2*_*H→Q*_ strain did not further impact the expression of these target genes. Taken together, all of these results demonstrated that the LadS pathway solely utilizes the H2 domain of GacS HK for signaling and that the histidine residue in position 859 of the GacSH2 is the cross point between the LadS and GacS signaling pathways.

### *In vitro* transphosphorylation of GacS H2 domain by LadS HK

**Fig 7:**
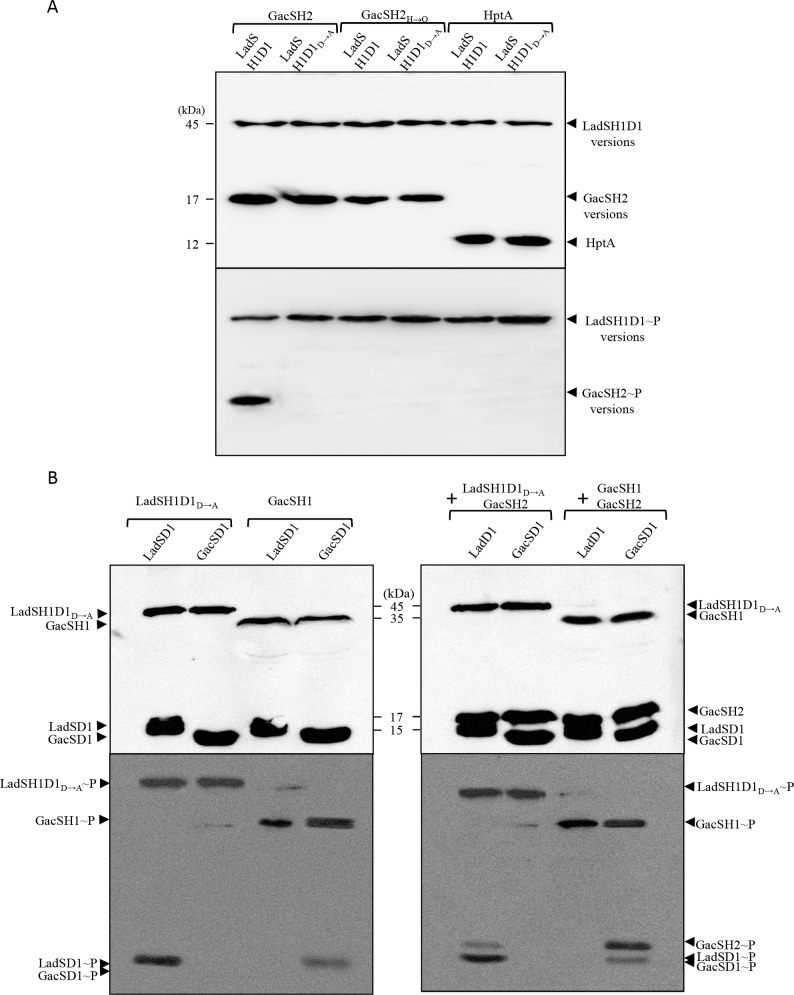
*In vitro* transphosphorylation assays. (A) For transphosphorylation assay between LadS or GacS variants and GacSH2 variants or HptA protein, 2 mM of LadSH1D1 or LadSH1D1_D→A_ recombinant proteins were incubated with [γ-^32^P] ATP and GacSH2 (lanes 1 and 2), GacSH2_H→Q_ (lanes 3 and 4) or HptA (lanes 5 and 6) at room temperature for 20 min (see Materials and Methods) then separated in an SDS-polyacrylamide gel in duplicate. (B) Transphosphorylation assay between the LadSH1D1_D→A_ or GacSH1 and LadSD1 or GacsD1 domains with or without the GacSH2 domain. Two mM of LadSD1 or GacSD1 recombinant proteins were incubated with [γ-^32^P] ATP and LadSH1D1_D→A_ or GacSH1 (left panel**)** together with the GacSH2 domain (right panel) at room temperature for 20 min. In both experiments mixtures of proteins were separated in an SDS-polyacrylamide gel in duplicate. Numbers on the left side are molecular weight standards (kDa). Locations of the recombinant proteins are indicated by arrowheads. For each experiment presented in panels A and B, one gel was detected by western blot using an anti-penta-His antibody (upper panel) while the other was autoradiographied (lower panel).

The results presented above strongly suggest that LadS HK could use the H2 domain of GacS HK to activate *rsm* gene transcription, probably via a transphosphorylation mechanism. To further confirm this, *in vitro* phosphorylation experiments were performed. The C-terminal His-tag forms of LadSH1D1, LadSH1D1_D→A_, GacSD1, LadSD1, GacSH1D1, GacSH2_,_ GacSH2_H→Q_ and HptA proteins were produced in *E*. *coli* and purified close to homogeneity from soluble fractions with nickel affinity columns and subjected to autophosphorylation assays by using [γ-^32^P]ATP (S4A and S4B Fig) (see Materials and Methods). LadSH1D1 and LadSH1D1_D→A_ proteins were found in an autophosphorylated form, while GacSH2_,_ GacSH2_H→Q_ and HptA were not (S4C Fig), confirming that LadSH1D1 and LadSH1D1_D→A_ are functional for their kinase activity. When mixed with LadSH1D1, GacSH2 was phosphorylated whereas GacSH2_H→Q_ and HptA were not (Fig 7A), demonstrating that LadS HK transphosphorylates the H2 domain of GacS HK on its H859 residue. LadSH1D1_D→A_ protein, while capable of autophosphorylation (S4C Fig), was unable to transphosphorylate the GacS H2 domain, demonstrating that D718 residue on D1 domain of LadS HK is fully required for the transphosphorylation process and that direct transphosphorylation cannot directly occur between the H1 domain of LadS HK and the H2 domain of GacS HK (Fig 7A).

**Fig 8:**
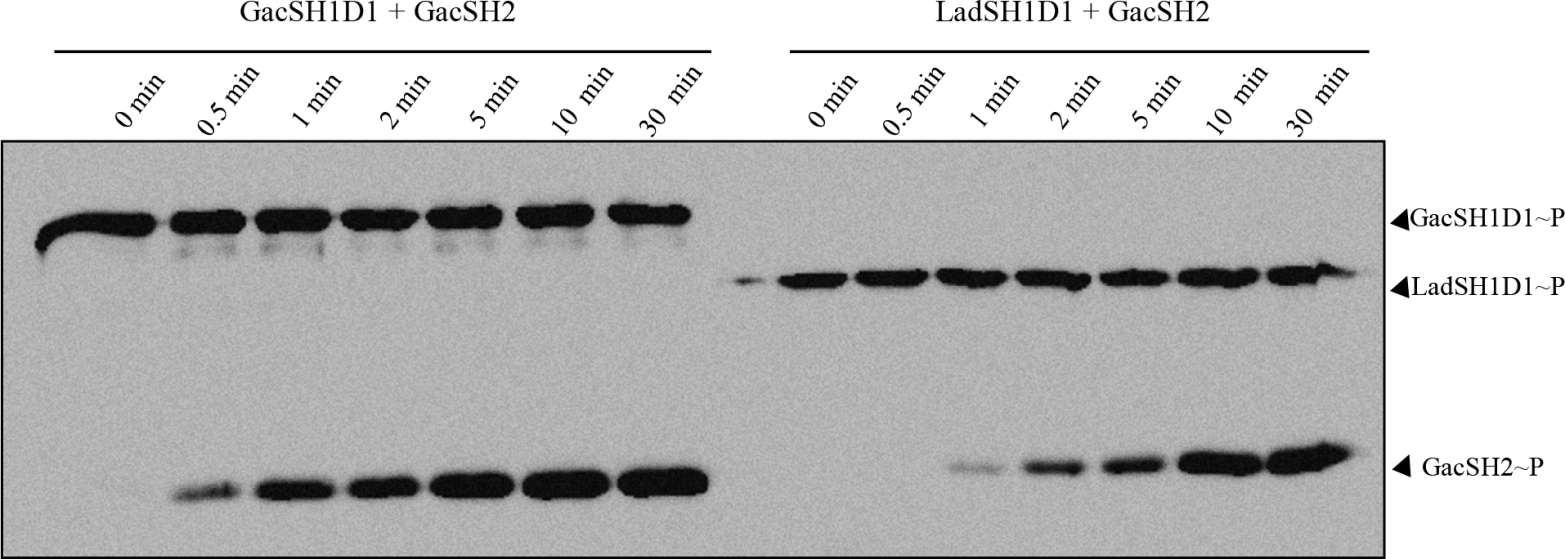
Transphosphorylation kinetic between the LadSH1D1 or GacSH1D1 and GacSH2 domains. Two mM of LadSH1D1 or GacSH1D1 and GacSH2 recombinant proteins were incubated with [γ-^32^P] ATP at room temperature. The reaction was stopped at different time points (see Materials and Methods) and the samples were separated in an SDS-polyacrylamide gel and autoradiographied.

To further exclude the crosstalk between LadSH1 and GacSD1 or between GacSH1 and LadSD1 domains, we produced the corresponding isolated domains (GacSH1, GacSD1, LadSD1) (S4B Fig). No phosphotransfer could be observed from the H1 domain of LadS to the D1 domain of GacS or from the H1 domain of GacS to the D1 domain of LadS. Meanwhile, phosphotransfer occurred between the H1 domain of LadS and the D1 domain of LadS and between the H1 domain of GacS and the D1 domain of GacS (Fig 7B, left panel). When GacSH2 was further added, we observed that the GacSH2 domain can only receive phosphate for the LadSH1D1_Q_→_A_/LadSD1 domain and for the GacSH1/GacSD1 domain combinations (Fig 7B, right panel), thereby finally proving that LadS signaling is H1_LadS_→D1_LadS_→H2_GacS_→D2_GacA._ From this last experiment, it clearly appeared that GacSH2 phosphorylation was less effective through LadS HK than through GacS HK. As the sampling was done at the same time in both experiments, this strongly suggests that the GacS signaling is faster than the LadS signaling. To confirm this observation, we performed kinetic experiments and clearly observed that phosphotransfer to the GacSH2 domain occurred 0.5–1 min earlier with the GacSH1D1 domain compared to the LadSH1D1 domain as a phosphodonor. This result suggests that the LadS hybrid HK forms a multicomponent signal transduction pathway with the GacS/GacA TCA and adds a supplementary level of regulation triggering chronic infection (Fig 8).

## Discussion

In the present study, we demonstrated that in *P*. *aeruginosa* the LadS hybrid HK forms a multicomponent signal transduction system with the GacS/GacA TCS. This multicomponent signal transduction system involves an H1_LadS_→D1_LadS_→H2_GacS_→D2_GacA_ signaling pathway, first through a phosphorelay between the H1 and D1 domains of LadS HK, and thus a transphosphorylation of the H2 domain of GacS HK. This LadS signaling pathway triggers both *rsmY* and *rsmZ* gene expression, the two sole direct targets controlled by the RR GacA [18] and further targets such as biofilm formation, H1-T6SS and T3SS expression.

As suggested or observed in other very closely phylogenetically related species to *P*. *aeruginosa* such as *P*. *fluorescens* [29] but never demonstrated in *P*. *aeruginosa* [7,25], our results showed that LadS controls both *rsmY* and *rsmZ* gene expression. These results also highlight the fact that LadS signaling occurs through the GacS/GacA TCS, reinforcing the notion that LadS and RetS HK reciprocally regulate the virulence factors under the *rsm* gene dependency [23,25]. This reciprocal regulation of LadS and RetS converging on GacS seems to be specific to some pseudomonas species such as *P*. *aeruginosa* or *P*. *fluorescens* but not generalizable to all, since in *P*. *syringae* the LadS, GacS and RetS HK do not control the same targets. This is exemplified for T3SS whose LadS- and RetS-dependent control is GacS-independent in this bacterial species [26,27]. In *P*. *syringae*, the absence of D1 domain in LadS HK may account for this GacS-independent T3SS regulation [27], while the *P*. *aeruginosa* LadS version without its D1 domain is nonfunctional (S5 Fig). Thus, in *P*. *aeruginosa*, LadS HK may control T3SS through its D1 domain and GacS HK. Another example of the crucial involvement of the H1D1 subdomain of *P*. *aeruginosa* LadS HK in LadS signaling is provided by the observed specific and natural *ladS* mutation in the PA14 strain, a 49 nucleotide duplication that leads to possible frameshift and results in a truncated and nonfunctional LadS protein lacking H1 and D1 domains [34]. In PA14, this nonfunctional mutation of the *ladS* gene can be reversed by trans-complementation with the PAK *ladS* gene.

The RetS hybrid HK [16,35,36] control of GacS involves a heterodimer formation between their H1 domains, which leads to suppression of GacS autophosphorylation in a phosphorelay-independent manner [23]. RetS conserved phosphorelay residues have been found dispensable [23] or not fully required [30] to control GacS, probably depending on the genetic background used. Conversely, we demonstrated that LadS control of GacS does not involve such a heterodimer mechanism in *P*. *aeruginosa*. In *P*. *aeruginosa*, LadS and GacS do not form heterodimers through their H1 domains as proven here by: i) pull-down and two-hybrid experiments and ii) the simple requirement of the H2 domain of the GacS HK for LadS signaling through GacS. Thus, the interaction between LadS H1 and GacS H1 domains as a mechanism of signaling between LadS HK and GacS HK can be ruled out. The functional bridge (phosphorelay) between the D1 domain of LadS and the H2 domain of GacS requires a transient interaction that has been potentially evidenced in *P*. *fluorescens* with the full-length proteins [32] and that we weakly observed between the LadS D1 domain and the GacS H2 domain using a two-hybrid approach (S6A Fig). Undoubtedly, as demonstrated in the present study, LadS signaling involves a phosphorelay mechanism along GacS, ruling out GacS HK titration by LadS, as RetS does. Although RetS suppresses GacS autophosphorylation, we demonstrated that LadS transphosphorylates the GacS HK by a phosphorelay involving the H_428_ of H1_LadS_ domain → the D_718_ of the D1_LadS_ domain → the H_859_ of the H2_GacS_ domain. Thus, LadS HK and the GacS/GacA TCS certainly form a multicomponent signal transduction system as with the CblS and CblT/CblR in *B*. *cenocepacia* [4]. The LadS signaling through the GacS H2 domain was GacS H2 domain-specific since i) the LadS hybrid HK did not use any of the three Hpt proteins (HptA, HptB, HptC) or any of the H2 domains of the four other unorthodox HK encoded in the *P*. *aeruginosa* genome (RocS1, RocS2, PA4112, PA4982) as demonstrated for the control of *rsmZ* gene expression (S6B Fig), ii) no interaction was observed between the LadS D1 domain and any of the three Hpt proteins or the H2 domains of the four other unorthodox HK (RocS1, RocS2, PA4112, PA4982) encoded in the *P*. *aeruginosa* genome (S6A Fig), and iii) a weak but significant interaction was observed between the LadS D1 and GacS H2 domains (S6A Fig). This confirms that the LadS signaling pathway exclusively use the Hpt module (the H2 domain) of the GacS unorthodox HK to control its targets.

Once the GacS/GacA signaling pathway is activated upon a signal that remains to be identified, *P*. *aeruginosa* is engaged in a chronic infection lifestyle characterized by biofilm and H1-T6SS production and the shutting down of the T3SS. The multicomponent signal transduction system made of the LadS hybrid HK and the GacS/GacA TCS (Fig 1B) is therefore able to integrate at least two different signals, one from GacS and the other from LadS, which also remains to be identified. LadS contains a putative 7-transmembrane (7TMR) region anchoring the HK into the inner membrane and a periplasmic sensor domain (diverse intracellular signaling module extracellular 2, DISMED2), whose predicted fold exhibits a putative binding site, highly conserved in carbohydrate-binding modules (CBMs) [37]. The activity of the multicomponent transduction system described here and the subsequent output response were also certainly modulated by the ratio of kinase to phosphatase activity [38,39]. Phosphatase-like activity leading to catalyzed dephosphorylation of phospho-response regulators described for unorthodox and hybrid sensors by reverse phosphotransfer [40–42] can be embodied in the cognate sensor kinase itself [42], carried out by the response regulator itself [40] or by another partner protein [43]. Thus, whether phosphatase-like activity leading to GacA dephosphorylation is assumed by GacS, LadS, any other HK of the network, by itself or by a partner protein requires extensive additional studies.

Thus, *P*. *aeruginosa* probably shares with *P*. *fluorescens* but not with *P*. *syringae* a unique molecular switch controlling Rsm sRNA-dependent virulence made of a central unorthodox HK and three hybrid HKs among which LadS HK makes a unique multicomponent system with the TCS GacS-GacA. This multiple input system probably reflects the variability of the environmental conditions *P*. *aeruginosa* faces and may result in a range of gradations of chronic infection through the integration of variable environmental signals.

## Materials and Methods

### Bacterial strains, growth conditions and media

The bacterial strains and plasmids used in this study are described in Tables 1 and 2 and the oligonucleotides used in S1 Table. Strains were grown aerobically in Luria–Bertani (LB) broth or on LB agar at 37°C or 30°C. To visualize bacterial two-hybrid interactions on solid medium, LB agar plates supplemented with the chromogenic substrate X-gal (5-bromo-4-chloro-3-indolyl-β-D-galactopyranoside, 40 μg/mL), isopropyl β-D-1-thiogalactopyranoside (IPTG) (100 μM), ampicillin (Ap, 100 μg/mL) and kanamycin (Km, 50 μg/mL) were used. Plasmids were introduced into *P*. *aeruginosa* by electroporation or by triparental mating using the conjugative properties of pRK2013. The transformants were selected on *Pseudomonas* isolation agar. Antibiotics were used at the following concentrations for *Escherichia coli*: 50 μg/mL ampicillin, 50 μg/mL streptomycin and 15 μg/mL tetracycline. For *P*. *aeruginosa*, 500 μg/mL carbenicillin, 2,000 μg/mL streptomycin and 200 μg/mL tetracycline were used.

**Table 1:** Strains used in this study.

Strains	Relevant characteristics*	Source
***E*. *coli***		
TG1	K-12, Δ(lac-pro) supE thi hsdD5/F' traD36 proA^+^B^+^ lacI^q^ lacZΔM15	Lab collection
DH5α	*endA1 hsdR17 supE44 thi-1 recA1 gyrA relA1 Δ(lacZYA-argF)U169 deoR (phi 80lacZ Δ M15)*	Lab collection
BTH101	F- *cya-99 araD139 galE15 galK16 rpsL1* (Sm^r^) *hsdR2 mcrA1 mcrB1*	[45]
Top10F’	F’ (*lacI*^q^ Tn10 (Tet^R^)) *mrcA Δ(mrr-hsdRMS-mcrBC*) Φ80 *lacZ*Δ*M15* Δ*lacX74 recA1*	Invitrogen
CC118(λpir)	Host strain for pKNG101 replication, Δ(*ara-leu*) *araD* Δl*ac*X74 *galE galK phoA20 thi-1 rpsE rpoB argE*(Am) *recA1* Rf^R^ (λpir)	Lab collection
***P*. *aeruginosa***		
PAK	Wild-type	[48]
PAKΔ*rsmY*	PAK deletion mutant for *rsmY* gene	[24]
PAKΔ*rsmZ*	PAK deletion mutant for *rsmZ* gene	[22]
PAKΔ*rsmY*Δ*rsmZ*	PAK deletion mutant for *rsmY* and *rsmZ* genes	[22]
PAKΔ*gacS*	PAK deletion mutant for *gacS* gene	[22]
PAKΔ*gacA*	PAK deletion mutant for *gacA* gene	[22]
PAKΔ*hptA*	PAK deletion mutant for *hptA* gene	[22]
PAKΔ*hptB*	PAK deletion mutant for *hptB* gene	[22]
PAKΔ*hptC*	PAK deletion mutant for *hptC* gene	[22]
PAKΔ*hptA*Δ*hptB*Δ*hptC*	PAK deletion mutant for *hptA*, *hptB and hptC* genes	This study
PAKΔ*rocS1*	PAK deletion mutant for *rocS1* gene	[49]
PAKΔ*rocS2*	PAK deletion mutant for *rocS2* gene	[49]
PAKΔ*PA4112*	PAK deletion mutant for *PA4112* gene	This study
PAKΔ*PA4982*	PAK deletion mutant for *PA4982* gene	This study
PAK*attB*::*pelA-lacZ*	PAK strain with *pelA-lacZ* inserted at *attB* sites	This study
PAK*attB*::*exoS-lacZ*	PAK strain with *exoS-lacZ* inserted at *attB* sites	This study
PAK*attB*::*rsmY-lacZ*	PAK strain with *rsmY-lacZ* inserted at *attB* sites	This study
PAK*attB*::*rsmZ-lacZ*	PAK strain with *rsmZ-lacZ* inserted at *attB* sites	This study
PAKΔ*rsmYattB*::*pelA-lacZ*	PAKΔ*rsmY* strain with *pelA-lacZ* inserted at *attB* sites	This study
PAKΔ*rsmYattB*::*exoS-lacZ*	PAKΔ*rsmY* strain with *exoS-lacZ* inserted at *attB* sites	This study
PAKΔ*rsmZattB*::*pelA-lacZ*	PAKΔ*rsmZ* strain with *pelA-lacZ* inserted at *attB* sites	This study
PAKΔ*rsmZattB*::*exoS-lacZ*	PAKΔ*rsmZ* strain with *exoS-lacZ* inserted at *attB* sites	This study
PAKΔ*rsmY*Δ*rsmZattB*::*pelA-lacZ*	PAKΔ*rsmY*Δ*rsmZ* strain with *pelA-lacZ* inserted at *attB* sites	This study
PAKΔ*rsmY*Δ*rsmZattB*::*exoS-lacZ*	PAKΔ*rsmY*Δ*rsmZ* strain with *exoS-lacZ* inserted at *attB* sites	This study
PAKΔ*gacSattB*::*rsmY-lacZ*	PAKΔ*gacS* strain with *rsmY-lacZ* inserted at *attB* sites	This study
PAKΔ*gacSattB*::*rsmZ-lacZ*	PAKΔ*gacS* strain with *rsmZ-lacZ* inserted at *attB* sites	This study
PAKΔ*gacAattB*::*rsmY-lacZ*	PAKΔ*gacA* strain with *rsmY-lacZ* inserted at *attB* sites	This study
PAKΔ*gacAattB*::*rsmZ-lacZ*	PAKΔ*gacA* strain with *rsmZ-lacZ* inserted at *attB* sites	This study
PAKΔ*hptAattB*::*rsmZ-lacZ*	PAKΔ*hptA* strain with *rsmZ-lacZ* inserted at *attB* sites	This study
PAKΔ*hptBattB*::*rsmZ-lacZ*	PAKΔ*hptB* strain with *rsmZ-lacZ* inserted at *attB* sites	This study
PAKΔ*hptCattB*::*rsmZ-lacZ*	PAKΔ*hptC* strain with *rsmZ-lacZ* inserted at *attB* sites	This study
PAKΔ*hptA*Δ*hptB*Δ*hptCattB*::*rsmZ-lacZ*	PAKΔ*hptA*Δ*hptB*Δ*hptC* strain with *rsmZ-lacZ* inserted at *attB* sites	This study
PAK::*miniTn7*	PAKstrain with empty mini Tn7 construct; Gm^R^	This study
PAKΔ*gacS*::*miniTn7*	PAKΔ*gacS* strain with empty mini Tn7 construct; Gm^R^	This study
PAKΔ*gacS*::*miniTn7gacS*_*FL*_	PAKΔ*gacS* strain with *gacS* in a mini Tn7 construct; Gm^R^	This study
PAKΔ*gacS*::*miniTn7gacSH2*	PAKΔ*gacS* strain with *gacSH2*_*WT*_ in a mini Tn7 construct; Gm^R^	This study
PAKΔ*gacS*::*miniTn7gacSH2*_*H→Q*_	PAKΔ*gacS* strain with *gacSH2*_*H→Q*_ in a mini Tn7 construct; Gm^R^	This study
PAKΔ*ladS*	PAK deletion mutant for *ladS* gene	[25]
PAKΔ*ladSD1*	PAK deletion mutant for D1 domain of *ladS* gene	This study
PAK*ladSH1*_*H→Q*_*D1*	Punctual chromosomal mutant H428Q of *ladS* gene in PAK	This study
PAK*ladSH1D1*_*D→A*_	Punctual chromosomal mutant D718A of *ladS* gene in PAK	This study
PAK*gacSH1*_*H→Q*_	Punctual chromosomal mutant H293Q of *gacS* gene in PAK	This study
PAK*gacSH2*_*H→Q*_	Punctual chromosomal mutant H859Q of *gacS* gene in PAK	This study
PAK*gacSH1*_*H→Q*_,*H2*_*H→Q*_	Punctual chromosomal mutant H293Q and H859Q of *gacS* gene in PAK	This study
PAK*gacSH1*_*H→Q*_Δ*ladS*	Punctual chromosomal mutant H293Q of *gacS* gene and deletion mutant for *ladS* gene.	This study
PAK*gacSH1*_*H→Q*_,*H2*_*H→Q*_Δ*ladS*	Punctual chromosomal mutant H293Q and H859Q of *gacS* gene and deletion mutant for *ladS* gene.	This study

* Sm^R^, streptomycin resistance, Gm^R^ gentamicin resistance

**Table 2:** Plasmids used in this study.

Plasmids	Relevant characteristics*	Source
pLic03	Vector containing T7 promoter with pBR322 origin of replication, Km^R^	[50]
pLic03_ LadSD1	pLic03 carrying the Nt-6his tagged LadSD1 subdomain DNA region	This study
pLic03_ GacSH1D1	pLic03 carrying the Nt-6his tagged GacSH1D1 subdomain DNA region	This study
pLic03_ GacSH1	pLic03 carrying the Nt-6his tagged GacSH1 subdomain DNA region	This study
pLic03_ GacSD1	pLic03 carrying the Nt-6his tagged GacSD1 subdomain DNA region	This study
pJF_*hptA*	pJF119EH carrying the 6his tagged *hptA* DNA region	This study
pBBRMCS3	Broad host range plasmid, Tc^R^	[51]
pBBRMCS4	Broad host range plasmid, Ap^R^	[51]
pBBRladS	pBBRMCS4 carrying the *ladS* gene	[25]
pBBR*ladSH1D1*	pBBRMCS4 carrying the *ladSH1D1* subdomain DNA region	This study
pBBR*ladSH1*_*H→Q*_*D1*	pBBRMCS4 carrying the *ladSH1*_*H→Q*_*D1* subdomain DNA region	This study
pBBR*ladSH1D1*_*D→A*_	pBBRMCS4 carrying the *ladSH1D1*_*D→A*_ subdomain DNA region	This study
pBBR_FLAG-*ladSH1*	pBBRMCS3 carrying the FLAG tagged *ladSH1* subdomain DNA region	This study
pBBR_FLAG-*gacSH1*	pBBRMCS3 carrying the FLAG tagged *gacSH1* subdomain DNA region	This study
pBBR_FLAG-*retSH1*	pBBRMCS3 carrying the FLAG tagged *retSH1* subdomain DNA region	This study
PCR2.1	TA cloning vector for PCR products, *lacZα* ColE1 f1 *ori*, Ap^R^, Km^R^	Invitrogen
pCR2.1_Strep-*ladSH1*	pCR2.1 carrying the Strep tagged *ladSH1* subdomain DNA region	This study
pCR2.1_Strep-gacS*H1*	pCR2.1 carrying the Strep tagged *gacSH1* subdomain DNA region	This study
pCR2.1_Strep-*retSH1*	pCR2.1 carrying the Strep tagged *retSH1* subdomain DNA region	This study
pCR2.1_FLAG-*ladSH1*	pCR2.1 carrying the FLAG tagged *ladSH1* subdomain DNA region	This study
pCR2.1_FLAG-*gacSH1*	pCR2.1 carrying the FLAG tagged *gacSH1* subdomain DNA region	This study
pCR2.1_FLAG-*retSH1*	pCR2.1 carrying the FLAG tagged *retSH1* subdomain DNA region	This study
pCR2.1*ladSH1D1*	pCR2.1 carrying the carrying the *ladSH1D1* subdomain DNA region	This study
pCR2.1*H1*_*H→Q*_*D1*	pCR2.1 carrying the *ladSH1*_*H→Q*_*D1* subdomain DNA region	This study
pCR2.1*H1D1*_*D→A*_	pCR2.1 carrying the *ladSH1D1*_*D→A*_ subdomain DNA region	This study
pCR2.1*gacSH2*	pCR2.1 carrying the *gacSH2* subdomain DNA region	This study
pCR2.1intladS*H1*_*H→Q*_	pCR2.1 carrying the internal fragment *ladSH1*_*H→Q*_	This study
pCR2.1intladS*D1*_*D→A*_	pCR2.1 carrying the internal fragment *ladSD1*_*D→A*_	This study
pUC18-miniTn7	mini-Tn*7* base vector with MCS; for cloning of suitable selection markers or other functional and selectable elements Gm^R^	[52]
pUC18-miniTn7-*gacSH2*	pUC18-miniTn7 carrying the *gacSH2* subdomain DNA region	This study
pUC18-miniTn7-*gacSH2*_*H→Q*_	pUC18-miniTn7 carrying the *gacSH2*_*H→Q*_ subdomain DNA region	This study
pRK2013	Tra^+^ Mob^+^ Km^R^	Lab collection
pKNG101Δ*PA4112*	Mutator plasmid for *PA4112* deletion Sm^R^	This study
pKNG101Δ*PA4982*	Mutator plasmid for *PA4982* deletion Sm^R^	This study
pKNG101Δ*hptA*	Mutator plasmid for *hptA* deletion Sm^R^	[22]
pKNG101Δ*hptB*	Mutator plasmid for *hptB* deletion Sm^R^	[22]
pKNG101Δ*hptC*	Mutator plasmid for *hptC* deletion Sm^R^	[22]
pKNG*ladSH1*_*H→Q*_*D1*	Mutator plasmid for point mutation H428Q in *ladS* gene Sm^R^	This study
pKNG*ladSH1D1*_*D→A*_	Mutator plasmid for point mutation D718A in *ladS* gene Sm^R^	This study
pKNG*gacSH1*_*H→Q*_	Mutator plasmid for point mutation H293Q in *gacS* gene Sm^R^	This study
pKNG*gacSH2*_*H→Q*_	Mutator plasmid for point mutation H859Q in *gacS* gene Sm^R^	This study
pKΔS	Mutator plasmid for *ladS* deletion Sm^R^	[25]
miniCTX-*lacZ*	Tc^r^ *lacZ*^+^; self-proficient integration vector with *tet*, V-*FRT-attPMCS*, *ori*, *int*, and *oriT*	[51]
miniCTX-*rsmY-lacZ*	Promoter region of *rsmY* gene inserted into miniCTX-*lacZ*, Tc^R^	This study
miniCTX-*rsmZ-lacZ*	Promoter region of *rsmZ* gene inserted into miniCTX-*lacZ*, Tc^R^	This study
miniCTX-*pelA-lacZ*	Promoter region of *pelA* gene inserted into miniCTX-*lacZ*, Tc^R^	This study
miniCTX-*exoS-lacZ*	Promoter region of *exoS* gene inserted into miniCTX-*lacZ*, Tc^R^	This study
pUT18C	Two-hybrid plasmid, *cyaAT18* fusion, Ap^R^	[53]
pUT18C-*ladSH1*	Two-hybrid plasmid containing *cyaAT18-ladS H1* domain fusion	This study
pUT18C-*retSH1*	Two-hybrid plasmid containing *cyaAT18-retS H1* domain fusion	[23]
pUT18C-*gacSH1*	Two-hybrid plasmid containing *cyaAT18-gacS H1* domain fusion	[23]
pUT18C-*ladSD1*	Two-hybrid plasmid containing *cyaAT18-ladS D1* domain fusion	This study
pKT25	Two-hybrid plasmid, *cyaAT25* fusion, Km^R^	[53]
pKT25-*ladSH1*	Two-hybrid plasmid containing *cyaAT25*–*ladS H1* domain fusion	This study
pKT25-*retSH1*	Two-hybrid plasmid containing *cyaAT25*–*retS H1* domain fusion	[23]
pKT25-*gacSH1*	Two-hybrid plasmid containing *cyaAT25*–*gacS H1* domain fusion	[23]
pKT25-*gacSH2*	Two-hybrid plasmid containing *cyaAT25*–*gacS H2* domain fusion	[49]
pKT25-*rocS1H2*	Two-hybrid plasmid containing *cyaAT25*–*rocS1 H2* domain fusion	[49]
pKT25-*rocS2H2*	Two-hybrid plasmid containing *cyaAT25*–*rocS2 H2* domain fusion	[49]
pKT25-*hptA*	Two-hybrid plasmid containing *cyaAT25*–*hptA* gene fusion	This study
pKT25-*hptB*	Two-hybrid plasmid containing *cyaAT25*–*hptB* gene fusion	This study
pKT25-*hptC*	Two-hybrid plasmid containing *cyaAT25*–*hptC* gene fusion	This study
pKT25-*PA4112H2*	Two-hybrid plasmid containing *cyaAT25*–*PA4112 H2* domain fusion	This study
pKT25-*PA4982H2*	Two-hybrid plasmid containing *cyaAT25*–*PA4982 H2* domain fusion	This study

* Sm^R^, streptomycin resistance, Gm^R^ gentamicin resistance, TC^R^ tetracyclin resistance, Ap^R^ ampicillin, Km^R^ Kanamycin

### Transcriptional fusions

The *rsmY*, *rsmZ*, *pelA* and *exoS* promoter regions were amplified by PCR with PAK genomic DNA using appropriate oligonucleotide pairs (S1 Table) and corresponding PCR products were cloned into pCR2.1 vector (Invitrogen) by TA cloning. After DNA sequencing, each promoter region cloned into pCR2.1 vector was excised by *Hind*III/*BamH*I and inserted into the linearized miniCTX-*lacZ* vector [44], thereby generating miniCTX-*rsmY-lacZ*, miniCTX-*rsmZ-lacZ*, miniCTX-*pelA-lacZ* and miniCTX-*exoS-lacZ* constructs. These plasmids were introduced in the different *P*. *aeruginosa* strains and site-specific recombination at the *attB* site, generating chromosomal *rsmY-lacZ*, *rsmZ-lacZ*, *pelA-lacZ* and *exoS-lacZ* fusions. The FRT cassette-excision step was performed, resulting in the generation of strains without tetracycline resistance.

### Truncated versions of LadS and GacS HK

DNA fragments corresponding to the cytoplasmic LadS1D1 part of the LadS hybrid HK (H1 and D1 subdomains), the LadSD1 domain, the cytoplasmic GacSH1D1 part of the GacS unorthodox HK (H1 and D1 subdomains), the GacSH1, the GacSD1 and the GacSH2 domains all fused to a His tag were amplified by PCR using appropriate oligonucleotide pairs (S1 Table). The DNA fragments corresponding to LadSH1D1 and GacSH2 were cloned into pCR2.1 vector yielding respectively pCR2.1*ladSH1D1* and pCR2.1*gacSH2* plasmids while LadSD1, GacSH1D1, GacSH1 and GacSD1 were cloned into pLic03 vector yielding pLic03_*ladSD1*, pLic03_*gacSH1D1*, pLic03_*gacSH1* and pLic03_*gacSD1*, respectively. After DNA sequencing, digestion was performed using *EcoR*I/*BamH*I for LadSH1D1 and *BamH*I/*Hind*III for GacSH2 for subcloning into pBBRMCS4 and pUC18-miniTn7 vectors, respectively, yielding pBBRLadSH1D1, referred to as LadSH1D1, and pUC18-miniTn7-*gacSH2*, referred to as GacSH2. Site-directed mutations in the DNA sequence of LadSH1D1 and GacSH2 were introduced respectively into pCR2.1*ladSH1D1* and pCR2.1*gacSH2* plasmids by Quick exchange site-directed mutagenesis method. Briefly, the conserved histidine residue at position 428 and the conserved aspartate residue at position 718 of LadS HK were changed into glutamine and alanine residues, respectively generating LadSH1_H→Q_D1 and LadSH1D1_D→A_ variants. The conserved histidine residue at position 859 of GacS HK was changed into glutamine, leading to the GacSH2_H→Q_ variant. This was done by using pCR2.1*ladSH1D1* and pCR2.1*gacSH2* vectors as matrices and by PCR using *pfu* turbo DNA polymerase (Stratagene) and 39-mer primers that incorporated appropriate mismatches to introduce the expected mutations (S1 Table). The resulting PCR products were digested with *Dpn*I for 1 hour. After DNA sequencing, each DNA sequence corresponding to LadSH1_H→Q_D1 and LadSH1D1_D→A_ or GacSH2_H→Q_ variants cloned into pCR2.1 were released by *EcoR*I/*BamH*I or *BamH*I/*Hind*III digestion, respectively, and inserted into pBBRMCS4 or pUC18-miniTn7, respectively, to generate pBBR*ladSH1*_*H→Q*_*D1* and pBBR*ladSH1D1*_*D→A*_ and pUC18-miniTn7-*gacSH2*_*H→Q*_.

### Construction of deletion mutants

PCR was used to generate a 500 bp DNA fragment upstream (Up) and a 500 bp DNA fragment downstream (Dn) of the PA4112 and PA4982 and of the LadSD1 domain of the *ladS* gene using the appropriate pairs of primers (S1 Table). Each PCR upstream and downstream product was linked together by overlapping PCR and products were cloned into pCR2.1. The linked DNA fragment was digested with *Xba*I and *Spe*I and cloned in the suicide vector pKNG101, yielding pKNG101Δ*PA4112* and pKNG101Δ*PA4982*, respectively. The suicide plasmids were introduced into *P*. *aeruginosa* via a three-partner procedure and the deletion mutants were obtained by double selection on LB agar supplemented with Irgasan (25 μg/mL) and streptomycin (1000 μg/mL) at 37°C and NaCl-free LB agar containing 6% sucrose at 30°C. The PAKΔhpAtΔ*hptB*Δ*hptC* triple mutant was constructed as follows: the *hptA* mutator cloned into the suicide pKNG101 vector [22] was introduced by mating into the PAKΔhptB leading to PAKΔhptAΔ*hptB*. The *hptC* mutator cloned into the suicide pKNG101 vector was further introduced by mating into the PAKΔhptAΔ*hptB* strain leading to the *PAKΔ*hptAΔ*hptB*Δ*hptC* strain. The *PAK*gacS*H1*_*H→Q*_*ΔladS* and *PAK*gacSH1_*H→Q*_*H2*_*H→Q*_*ΔladS* strains were constructed by introducing the pKNG101Δ*ladS* vector by mating into the *PAK*gacSH1_*H→Q*_ and the PAK*gacSH1*_*H→Q*_*H2*_*H→Q*_ strains, respectively.

### Construction of chromosomal punctual mutants

For engineering strains: 1) PAK*ladSH1*_*H→Q*_*D1* and PAK*ladSH1D1*_*D→A*_ harboring in their chromosomal copy of *ladS* gene a point mutation of the conserved histidine residue at position 428 or of the conserved aspartate residue at position 718, respectively, and 2) PAK*gacSH1*_*H→Q*_ and PAK*gacSH1*_*H→Q*_ *H2*_*H→Q*_ harboring in their chromosomal copy of *gacS* gene a point mutation of the conserved histidine at position 293 or of both conserved histidine residues at positions 293 and 859, respectively, the upstream and downstream sequences (approximately 500 bp) were amplified from PAK genomic DNA using the appropriate pairs of primers (S1 Table). Each PCR upstream and downstream product was linked together by overlapping PCR. For the *ladS* and the *gacSH2*_*H→Q*_ variants, the PCR products were cloned into pCR2.1. Then, each linked DNA fragment was digested with *Xba*I and *Spe*I and cloned into the suicide vector pKNG101, yielding pKNG*ladSH1*_*H→Q*_*D1*, pKNG*ladSH1D1*_*D→A*_ and pKNG*gacSH2*_*H→Q*_, respectively. For the *gacSH1*_*H→Q*_ variant, the PCR product was digested with *Apa*I and *Spe*I and cloned into the suicide vector pKNG101, yielding pKNG*gacSH1*_*H→Q*_. The suicide plasmids were introduced into the PAK strain and a double recombination event was selected using NaCl-free LB plates supplemented with 6% sucrose. The presence of mutations was checked by sequencing. For the *gacSH1*_*H→Q*_*H2*_*H→Q*_ variant, the pKNG*gacSH1*_*H→Q*_ suicide vector was further introduced by mating into the PAK*gacSH2*_*H→Q*_ strain to further obtain after the double recombination event the PAK*gacSH1*_*H→Q*_*H2*_*H→Q*_ strain.

### Biofilm assay

The *P*. *aeruginosa* adherence assay was performed in individual glass tubes containing 1 mL of medium as described previously [22]. After 5 hours of incubation at 30°C, the cultures were incubated with 1% Crystal Violet for 10 min and washed twice. Staining was extracted by treatment with 400 μL 95% ethanol. Subsequently, 600 μL of water was added and OD_570nm_ was measured. All quantification assays were performed at least in triplicate.

### Bacterial two-hybrid experiments

The DNA regions encoding the HptA, HptB and HptC proteins, the H1 and D1 domains of LadS HK and the H2 domains of PA4112 and PA4982 HKs were PCR amplified by using PAK genomic DNA with appropriate oligonucleotide pairs (S1 Table). PCR product of HptA was digested by *Pst*I/KpnI, while PCR products of HptB, HptC and H2 domains and of PA4112 and PA4982 HKs were digested by *KpnI/XbaI* and cloned into pKT25, yielding pKT25-*hptA*, pKT25-*hptB*, pKT25-*hptC*, pKT25-*PA4112H2* and pKT25-*PA4982H2*, respectively. PCR product of the D1 domain of LadS HK was digested by *PstI*/*ClaI* and cloned into pUT18C, yielding pUT18C-*ladSD1*. The DNA region encoding the H1 domain of LadS HK was digested by *Xba*I/*Sac*I and cloned into pKT25 or pUT18C vectors, yielding pKT25-*ladSH1* and pUT18C-*ladSH1*, respectively. The adenylate cyclase-deficient *E*. *coli* strain BTH101 was used to screen for positive interactions [45,46]. BTH101 competent cells were transformed simultaneously with pKT25 and pUT18C derivatives and transformants were selected on agar plates supplemented with ampicillin (100 μg/mL) and kanamycin (50 μg/mL). Single colonies were spotted on solid medium, LB-agar plates supplemented with the chromogenic substrate X-gal (5-bromo-4-chloro-3-indolyl-β-D-galactopyranoside, 40 μg/mL), isopropyl β-D-1-thiogalactopyranoside (IPTG) (100 μM), ampicillin (100 μg/mL) and kanamycin (50 μg/mL). Positive interactions were identified as blue colonies after 24 hours’ incubation at 30°C. The co-transformants of interest were grown in liquid LB medium at 37°C at 250 rpm for 16 hours. Five hundred μL of each culture was pelleted and mixed with 900 μL of Z buffer (10.7 g l^−1^ Na_2_HPO_4_ 2H_2_O, 5.5 g l^−1^ NaH_2_PO_4_, 0.75 g l^−1^ KCl, 0.246 g l^−1^ MgSO_4_.7H_2_O, 2.7 mL l^− 1^β-mercaptoethanol, pH 7) before addition of 20 μL of 0.1(w/v) SDS and 100 μL of CHCl_3_ for permeabilization. A 40 μL volume of orthonitrophenyl-β-galactoside (ONPG) solution (4 mg/mL in Z buffer without β-mercaptoethanol) was added to 20 μL of permeabilized cells diluted in 180 μL of Z buffer, and β-galactosidase activity was then calculated and expressed in Miller units.

### Pull-down experiments

A DNA fragment corresponding to H1 domains of GacS HK, RetS HK and LadS HK was amplified by PCR with an N-terminal FLAG or Strep tag (see S1 Table) and cloned in pCR2.1. The sequences of each construction were checked by sequencing and the DNA fragment of each H1 domain with FLAG tag was further digested with *Xba*I/*Sac*I (LadS HK, RetS HK) or *Pst*I/*Sac*I (GacS HK) for subcloning into pBBRMCS3 vector. Production of each H1 FLAG- or Strep-tagged domain was checked in *E*. *coli* TG1 cells. The 16 following combinations were further examined: pBBR_FLAG-*retSH1*/pCR2.1_Strep*-retSH1*, pBBR_FLAG-*ladSH1*/pCR2.1_Strep-*retSH1*, pBBR_FLAG-*gacSH1*/pCR2.1_Strep-*retSH1*, pBBRMCS3/pCR2.1_Strep-*retSH1*, pBBR_FLAG-*retSH1*/pCR2.1_Strep-*ladSH1*, pBBR_FLAG-*ladSH1*/pCR2.1_Strep-*ladSH1*, pBBR_FLAG-*gacSH1*/pCR2.1_Strep-*ladSH1*, pBBRMCS3/pCR2.1_Strep-*ladSH1*, pBBR_FLAG-*retSH1*/pCR2.1_Strep-*gacSH1*, pBBR_FLAG-*ladSH1*/pCR2.1_Strep-*gacSH1*, pBBR_FLAG-*gacSH1*/pCR2.1_Strep-*gacSH1*, pBBRMCS3/pCR2.1_Strep-*gacSH1*, pBBR_FLAG-*retSH1*/pCR2.1, pBBR_FLAG-*ladSH1*/pCR2.1, pBBR_FLAG-*gacSH1*/pCR2.1 and pBBRMCS3/pCR2.1. Thirty milliliters of cell cultures at OD_600nm_ 0.6 were cultured with 1 mM IPTG for 3 hours. Forty OD units of cells were harvested and resuspended in 2mL of 10 mM Tris buffer pH 8.0 supplemented with cOmplete, EDTA-free Protease Inhibitor Cocktail (Roche) and 100 mM NaCl. Ten μg/mL of DNase and RNase were added and cells were lysed by sonication. Total lysates were mixed with 50 μL of agarose beads coupled with antibody against Strep peptide (Streptactin Superflow IBA) and incubated on a rotating wheel for 1 hour at 4°C. The unbound fraction was collected by centrifugation for 2 min at 2000 rpm. Beads were washed three times with 1 mL of 10 mM Tris 50 mM NaCl, and were collected by centrifugation, resuspended in loading buffer and heated for 10 min at 95°C before analysis by SDS-PAGE and immunoblotting.

### Overexpression and purification of proteins

Recombinant His-tagged LadSH1D1, LadSH1D1_D→A_, LadSD1, GacSH1D1, GacSH1, GacSD1, GacSH2, GacSH2_H→Q_ and HptA proteins were purified from soluble extracts of the TG1 strain containing either pJF_*ladSH1D1*, pJF_*ladSH1D1*_*D→A*_, pJF_*gacSH2*, pLic03_ *gacSH1D1*, pLic03_ *gacSH1*, pLic03_*gacSD1*, pLic03_*ladSD1*, pJF_*gacSH2*_*H→Q*_ or pJF_*hptA*. Cultures were grown aerobically at 37°C until OD_600nm_ 0.6 and induced for 3 hours with 1 mM IPTG for recombinant proteins produced by the pJF119EH vector or 250 μM IPTG for recombinant proteins produced by the pLic03 vector. A one-step purification via affinity chromatography was facilitated by the presence of a His_Tag at the C-terminal extremity of LadSH1D1, LadSH1D1_D→A_, GacSH2, GacSH2_H→Q_ and HptA and at the N-terminal extremity of LadSD1, GacSH1D1, GacSH1 and GacSD1, using nickel columns (HiTrap HP chelating column) as described by the manufacturer (GE healthcare). Proteins were eluted in an imidazole gradient buffer (20 mM to 500 mM) and analyzed by SDS-PAGE.

### *In vitro* phosphorylation assay

Evidence of a phosphotransfer between LadS variants (LadSH1D1 and LadSH1D1_D→A_) or GacS variants (GacSH1D1 and GacSH1) and GacSH2 variants (GacSH2 and GacSH2_H→Q_), GacSD1 domain, LadSD1 domain or HptA protein was tested by *in vitro* phosphorylation assays. These assays were carried out in 10 μL of reaction buffer (50 mM Tris-HCl [pH 7.6], 50 mM KCl, 5 mM MgCl_2_, 1 mM dithiothreitol containing 0.1 mM [γ-^32^P] ATP) with 2 mM of purified proteins, and the mixture was incubated at room temperature for 20 min. The reaction was stopped by adding 5 μl of loading buffer (120 mM Tris–HCl (pH 8.8), 3.5 mM EDTA, 0.6 M sucrose, 0.06% (w/v) bromophenol blue, 6% (w/v) SDS, 0.1 M DTT and 1.6% (v/v) β-mercaptoethanol). All samples were analyzed by SDS-PAGE and radioactivity was revealed 12 hours after exposition by using a PhosphorImager screen (Molecular Dynamics).

### Phosphotransfer kinetic experiments

The phosphotransfer kinetic between the LadSH1D1 or GacSH1D1 and GacS H2 domains was further followed *in vitro*. These assays were conducted as follows: 2 mM of purified LadSH1D1 or GacSH1D1 proteins was incubated at room temperature for 20 min in 10 μL of reaction buffer (50 mM Tris-HCl [pH 7.6], 50 mM KCl, 5 mM MgCl_2_, 1 mM dithiothreitol containing 0.1 mM [γ-^32^P] ATP). Then 2 mM of purified GacSH2 protein was added and the transphosphorylation reaction was stopped after 0, 0.5, 1, 2, 5, 10 or 30 min by adding 5 μl of loading buffer as described above. All samples were analyzed by SDS-PAGE and radioactivity was revealed 10 hours after exposition by using a PhosphorImager screen (Molecular Dynamics).

### Western blot

Bacterial cell pellets were resuspended in loading buffer (Tris HCl 0.1M pH 8.8; EDTA 3mM; Saccharose 0.6M; SDS 6%; DTT 0.1 M; β-mercaptoethanol 1.5% bromophenol blue 0.03%). The samples were boiled and separated in SDS gels containing 20% acrylamide and blotted onto nitrocellulose membranes. After overnight saturation at 4°C in phosphate-buffered saline (PBS), 0.1% Tween 20 and 10% skimmed milk, the membrane was incubated for 1 hour in PBS 0.1% Tween 20, and 10% skimmed milk with appropriated antibodies (1:5,000 for pentaHis; 1:2,000 anti-VgrG1a and 1:2,500 for anti-Hcp1), washed three times with PBS 0.1% Tween 20, incubated for 1 hour in PBS 0.1% Tween 20, and 10% skimmed milk with anti-mouse conjugate HRP antibody (Sigma) (1:5,000), washed three times with PBS 0.1% Tween 20 and then revealed with a Super Signal Chemiluminescence system (Pierce).

### Measurements of β-galactosidase activity

Strains carrying the *lacZ* transcriptional fusions were grown in LB under agitation at 37°C. The bacterial cells were collected by centrifugation at different growth times. The β-galactosidase activity was measured using the method of Miller. Experiments with strains carrying the *exoS-lacZ* fusion were performed similarly except that EGTA (5 mM) and MgCl_2_ (20 mM) were added in the growth medium.

### RT-qPCR

The PAKΔ*gacS*::*miniTn7gacSH2* and PAKΔ*gacS*::*miniTn7gacSH2*_*H→Q*_ strains that had received the pBBR*ladS* plasmid or the corresponding pBBRMCS4 empty vector and the PAK, PAKΔ*ladS*, PAK*ladSH1*_*H→Q*_*D1* and PAK*ladSH1D1*_*D→A*_ strains were grown in the presence of EDTA at 37°C under agitation until OD_600nm_ reached 4. Total cellular RNA from 10 mL of cultures was isolated, using the PureYield RNA Midiprep System (Promega), cleaned up and concentrated using the RNeasy kit (Qiagen). The yield, purity and integrity of RNA were further evaluated on Nanodrop and Experion devices. Reverse transcription was performed on 2 μg of RNA by using the SuperScript III first-strand synthesis system (Invitrogen). Real-time PCR runs were carried out on a CFX96 Real-Time System (Bio-Rad). Cycling parameters of the real-time PCR were 98°C for 2 min, followed by 45 cycles of 98°C for 5 s and 60°C for 10 s, ending with a melting curve from 65°C to 95°C to determine the specificity of the amplification. To determine the amplification kinetics of each product, the fluorescence derived from the incorporation of EvaGreen into the double-stranded PCR products was measured at the end of each cycle using a SsoFast EvaGreen Supermix 2X Kit (Bio-Rad). The results were analyzed using Bio-Rad CFX Manager Software 3.0 (Bio-Rad). The *uvrD* gene was used as a reference for normalization, in particular because transcription of *uvrD* is fairly stable in bacteria exposed to antibiotics even at relatively high concentrations [47].

## Supporting Information

S1 Fig (A) Activities of the *rsmY–lacZ* (blue circles) and *rsmZ–lacZ* (brick-red-colored triangles) transcriptional chromosomal fusions were monitored at different growth stages in the PAK strain, which had received the pBBRMCS4 (empty symbols) or the pBBR*ladS* (filled symbols) vectors. Corresponding β-galactosidase activities are expressed in Miller units and correspond to mean values (with error bars) obtained from three independent experiments. (B) RsmY (blue bars), RsmZ (brick-red-colored bars), VgrG1 (green bars), PelA (violet bars) and ExoS (royal blue bars) transcript levels were monitored using RT-qPCR and fold induction was presented in the strains PAK, PAKΔ*ladS*, PAKpBBRMCS4 and PAKpBBR*ladS*. Moderated t-tests were performed and *, **, *** and ns referred to p<0.05, p<0.01 and p<0.001 and nonsignificant difference, respectively. (C) Number of LadS mRNA copies were expressed per μg of total RNA retrotranscribed in PAK, PAKΔ*ladS*, PAK + pBBRMCS4 and PAK + pBBR*ladS* vectors. Mean values (with error bars) were obtained from three independent experiments. Wilcoxon-Mann-Whitney tests were performed and *** referred to p<0.001.
(TIF)

S2 Fig Co-production of N-terminal FLAG or Strep versions of H1 domain of GacS, LadS and RetS HKs in *E*. *coli*. Production of each FLAG- or Strep-tagged proteins was detected in whole cell extracts using western blot using StrepTactin Alkaline Phosphatase conjugate (upper panel) and anti-FLAG antibody detection (lower panel).
(TIF)

S3 Fig Domain organization of GacS and LadS HK and map of constructs used for expression experiments of the present study. (TIF)

S4 Fig Purification and phosphorylation assays of LadSH1D1, LadSH1D1_D→A_, GacSH2, GacSH2_H→Q_ and HptA recombinant proteins. Purified His-tagged forms of LadSH1D1, LadSH1D1_D→A_, GacSH2, GacSH2_H→Q_, HptA (A), LadSD1, GacSH1D1, GacSD1 and GacSH1 (B) proteins separated in an SDS-polyacrylamide gel stained with coomassie blue (upper panel) or detected by western blot using an anti-penta-His antibody (lower panel). Numbers on the left side are molecular weight standards (kDa) and locations of the recombinant proteins are indicated by arrowheads. *In vitro* phosphorylation assays of LadSH1D1, LadSH1D1_D→A_, GacSH2, GacSH2_H→Q_, HptA (C), LadSD1, GacSH1D1, GacSD1 and GacSH1 (D) proteins. Each protein was incubated with [γ-^32^P] ATP at room temperature for 20 min (see materials and methods) then resolved by an SDS-polyacrylamide gel and autoradiographied. Locations of the recombinant proteins are indicated by arrowheads.
(TIF)

S5 Fig Role of the LadS D1 domain in the LadS signaling pathway. Transcript levels of RsmY (blue bars) and RsmZ (brick-red-colored bars) were monitored in the PAK, PAKΔ*ladS* and PAKΔ*ladSD1* strains using RT-qPCR and fold induction was presented for the two mutant strains as compared to the PAK strain. Moderated t-tests were performed; *, ** and *** referred respectively to p<0.05, p<0.01 and p<0.001.
(TIF)

S6 Fig Role of the free Hpt proteins and H2 domains of unorthodox HKs in the LadS signaling pathway. (A) The *hptA*, *hptB*, *hptC*, *rocS1H2*, *rocS2H2*, *PA4112H2*, *PA4982H2* and gac*SH2* DNA regions were cloned into the two-hybrid pKT25 and the *ladS*-D1 DNA region was cloned into pUT18C. All of the pKT25 construction as well as the empty vector were co-transformed in BTH101 cells with pUT18C vector containing or not *ladS*-D1 DNA regions and β-galactosidase activities were measured after 16 hours of growth. All experiments were carried out in at least triplicate, and error bars represent standard deviation. (B) The pBBR*ladS* plasmid containing the *ladS* HK gene (dark bars) and the pBBRMCS4 corresponding empty cloning vector (light bars) were conjugated in the PAK, PAKΔhptA, PAKΔhptB, PAKΔ*hptC*, PAKΔ*hptA*Δ*hptB*Δ*hpt*C, PAKΔrocS1, PAKΔ*rocS2*, PAKΔPA4112, PAKΔPA4982 and PAKΔ*gacS* strains. Activity of the *rsmZ–lacZ* (brick-red-colored) transcriptional chromosomal fusion was monitored after 6 hours of growth (OD_600nm_≈4) and corresponding β-galactosidase activities are expressed in Miller units and correspond to mean values (with error bars) obtained from three independent experiments.
(TIF)

S1 Table Oligonucleotides used in this study. (DOCX)
