# Supplementary figures and images for: The Hybrid Histidine Kinase LadS Forms a Multicomponent Signal Transduction System with the GacS/GacA Two-Component System in Pseudomonas aeruginosa
Source: PLoS Genet. 2016 May 13;12(5):e1006032. doi: 10.1371/journal.pgen.1006032 (PMC4866733; doi:10.1371/journal.pgen.1006032)

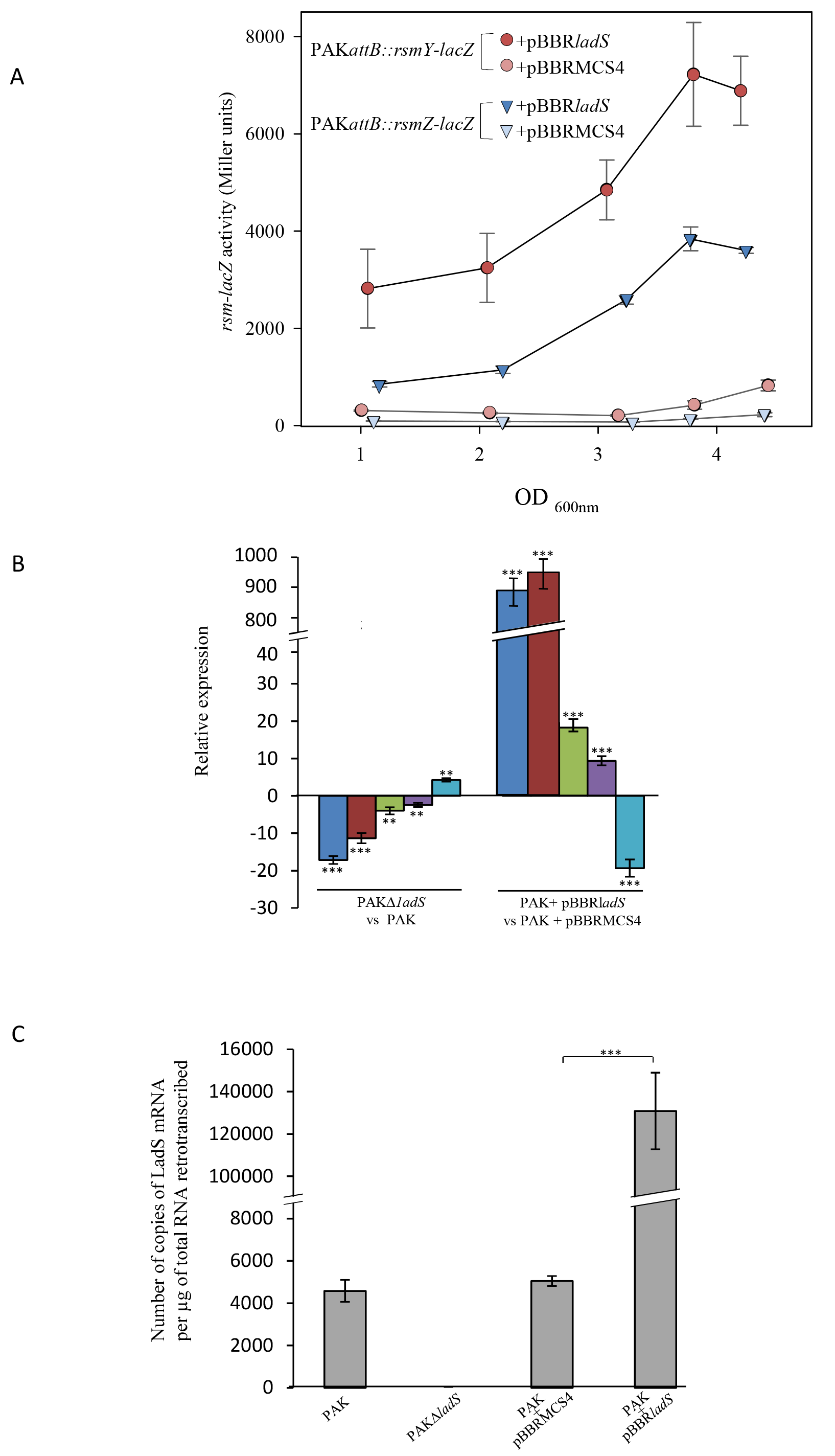

Supplement: S1 Fig — (A) Activities of the rsmY–lacZ (blue circles) and rsmZ–lacZ (brick-red-colored triangles) transcriptional chromosomal fusions were monitored at different growth stages in the PAK strain, which had received the pBBRMCS4 (empty symbols) or the pBBRladS (filled symbols) vectors. Corresponding β-galactosidase activities are expressed in Miller units and correspond to mean values (with error bars) obtained from three independent experiments. (B) RsmY (blue bars), RsmZ (brick-red-colored bars), VgrG1 (green bars), PelA (violet bars) and ExoS (royal blue bars) transcript levels were monitored using RT-qPCR and fold induction was presented in the strains PAK, PAKΔladS, PAKpBBRMCS4 and PAKpBBRladS. Moderated t-tests were performed and *, **, *** and ns referred to p<0.05, p<0.01 and p<0.001 and nonsignificant difference, respectively. (C) Number of LadS mRNA copies were expressed per μg of total RNA retrotranscribed in PAK, PAKΔladS, PAK + pBBRMCS4 and PAK + pBBRladS vectors. Mean values (with error bars) were obtained from three independent experiments. Wilcoxon-Mann-Whitney tests were performed and *** referred to p<0.001. (TIF) [file pgen.1006032.s001.tif]

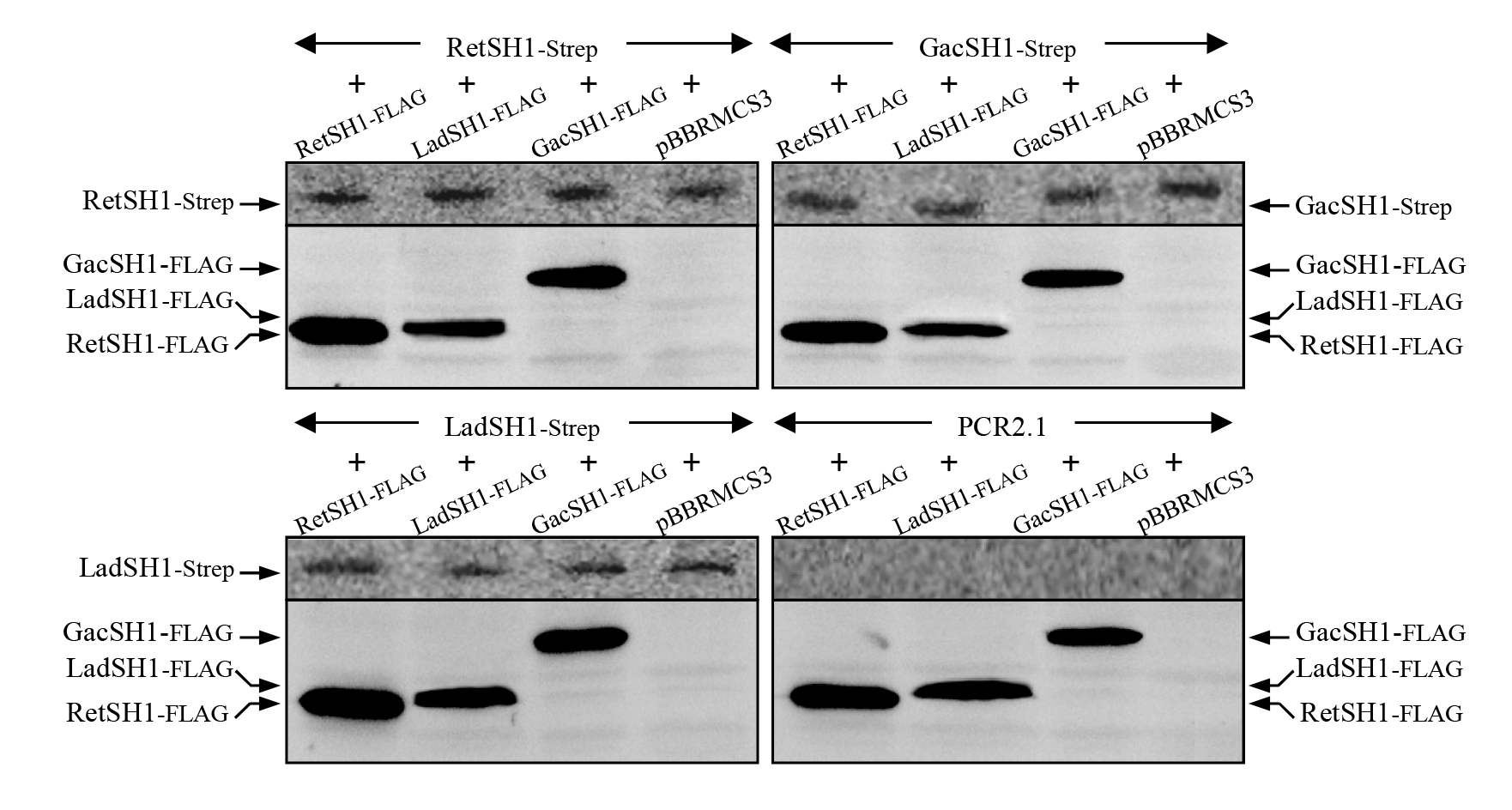

Supplement: S2 Fig — Production of each FLAG- or Strep-tagged proteins was detected in whole cell extracts using western blot using StrepTactin Alkaline Phosphatase conjugate (upper panel) and anti-FLAG antibody detection (lower panel). (TIF) [file pgen.1006032.s002.tif]

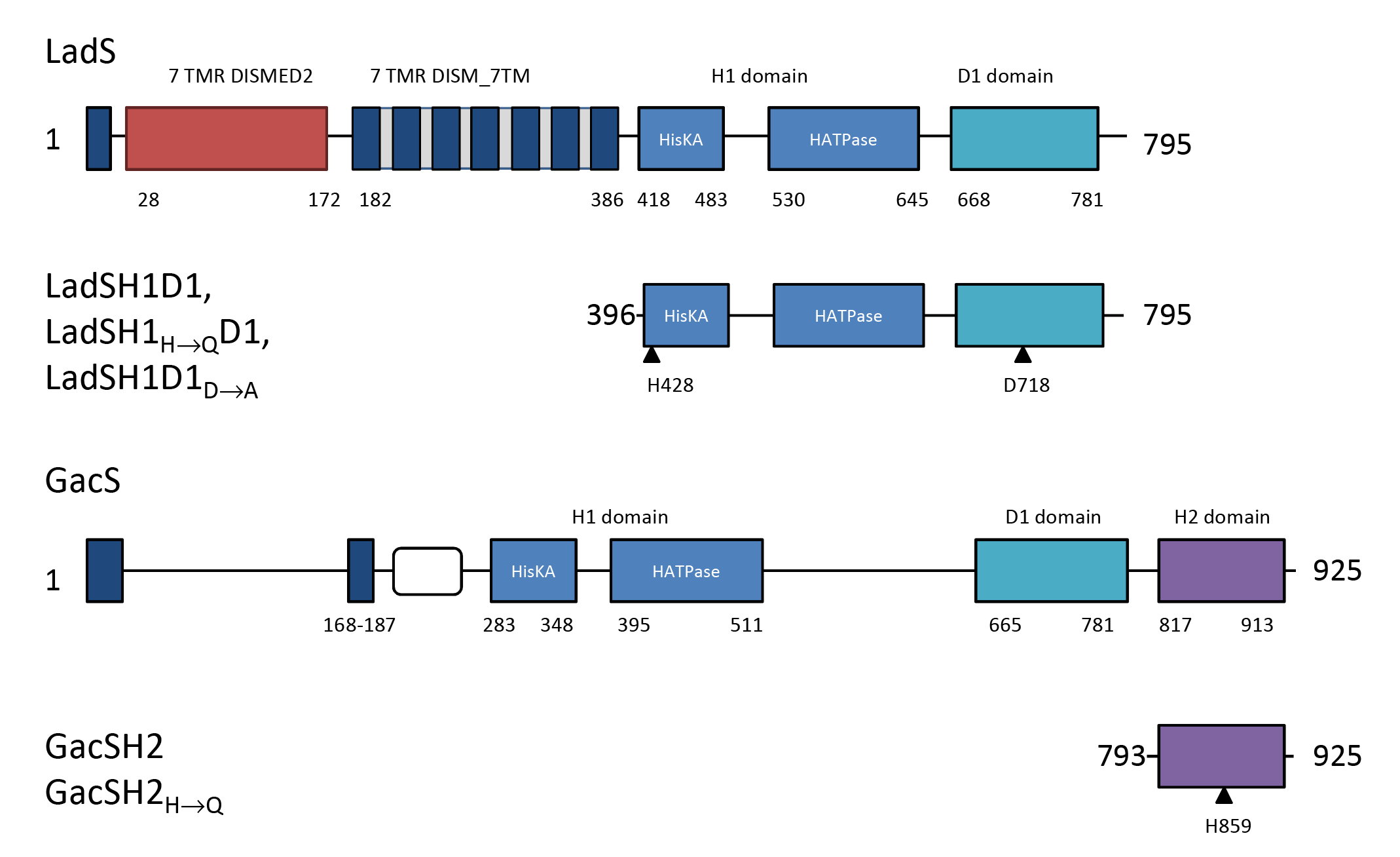

Supplement: S3 Fig — (TIF) [file pgen.1006032.s003.tif]

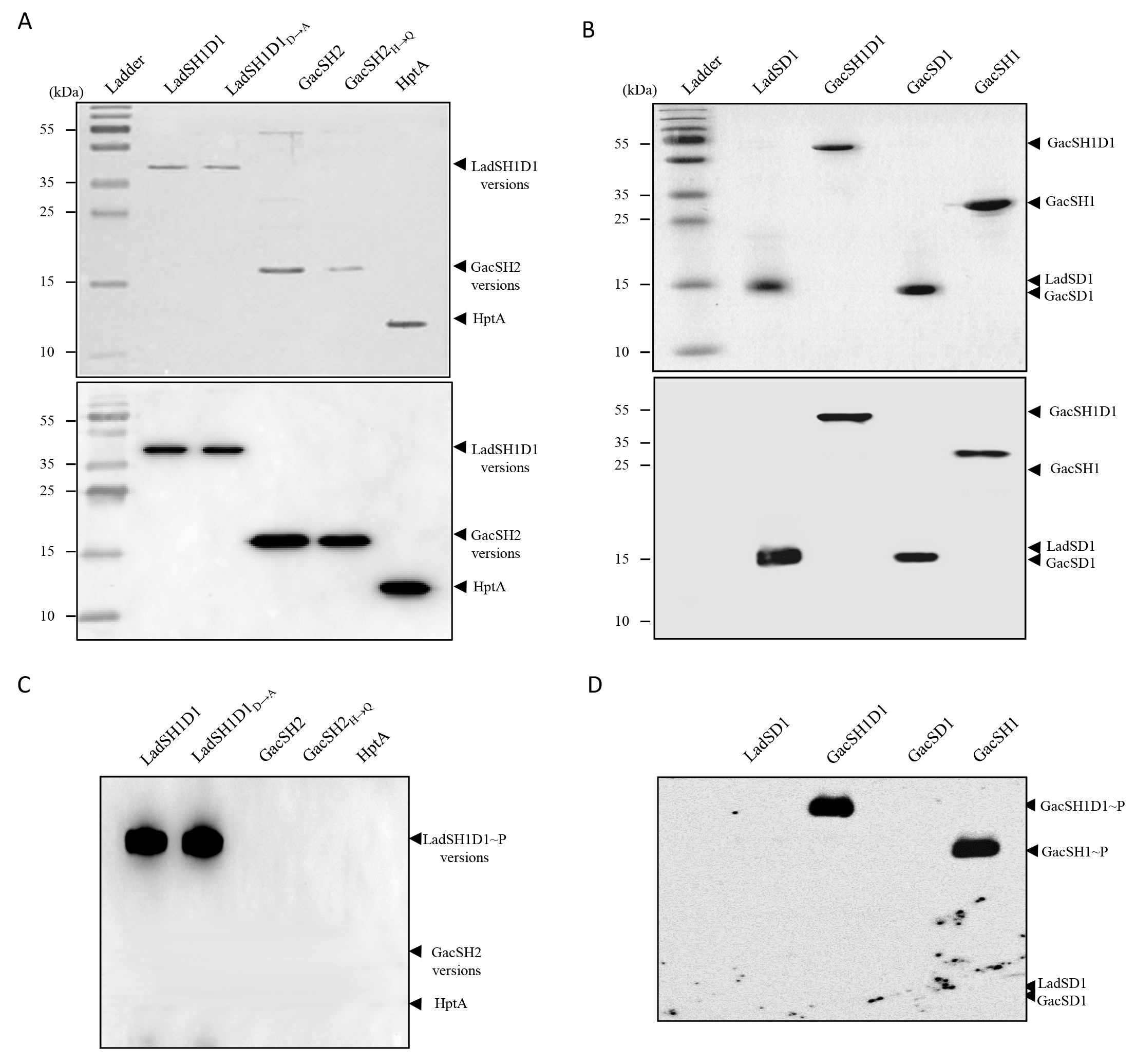

Supplement: S4 Fig — Purified His-tagged forms of LadSH1D1, LadSH1D1D→A, GacSH2, GacSH2H→Q, HptA (A), LadSD1, GacSH1D1, GacSD1 and GacSH1 (B) proteins separated in an SDS-polyacrylamide gel stained with coomassie blue (upper panel) or detected by western blot using an anti-penta-His antibody (lower panel). Numbers on the left side are molecular weight standards (kDa) and locations of the recombinant proteins are indicated by arrowheads. In vitro phosphorylation assays of LadSH1D1, LadSH1D1D→A, GacSH2, GacSH2H→Q, HptA (C), LadSD1, GacSH1D1, GacSD1 and GacSH1 (D) proteins. Each protein was incubated with [γ-32P] ATP at room temperature for 20 min (see materials and methods) then resolved by an SDS-polyacrylamide gel and autoradiographied. Locations of the recombinant proteins are indicated by arrowheads. (TIF) [file pgen.1006032.s004.tif]

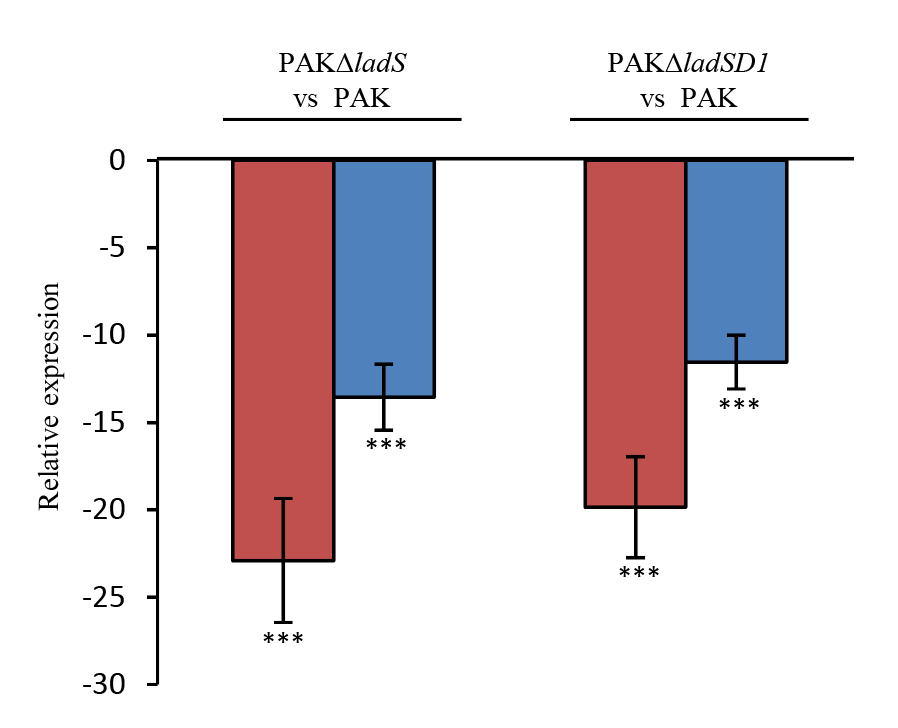

Supplement: S5 Fig — Transcript levels of RsmY (blue bars) and RsmZ (brick-red-colored bars) were monitored in the PAK, PAKΔladS and PAKΔladSD1 strains using RT-qPCR and fold induction was presented for the two mutant strains as compared to the PAK strain. Moderated t-tests were performed; *, ** and *** referred respectively to p<0.05, p<0.01 and p<0.001. (TIF) [file pgen.1006032.s005.tif]

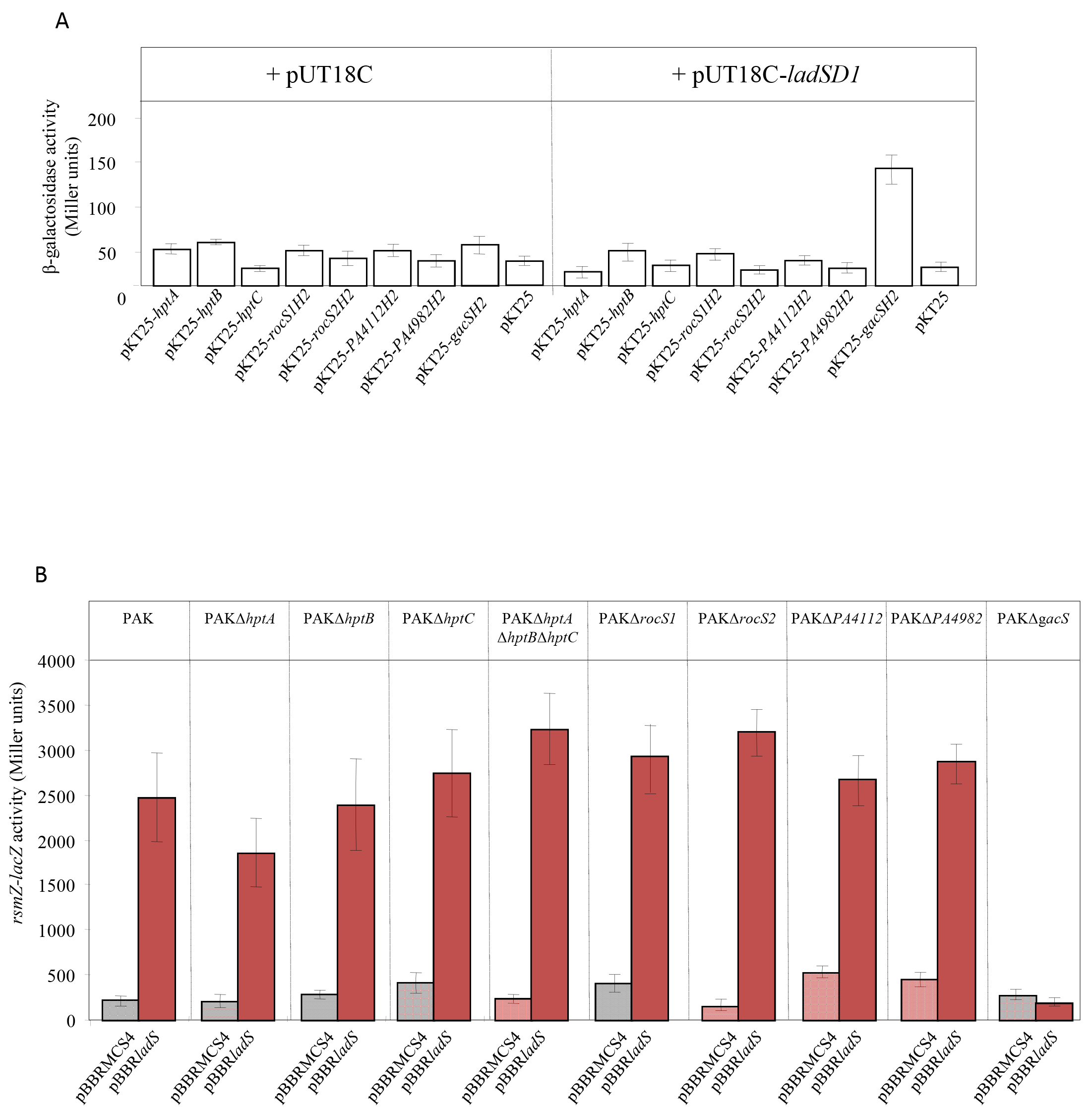

Supplement: S6 Fig — (A) The hptA, hptB, hptC, rocS1H2, rocS2H2, PA4112H2, PA4982H2 and gacSH2 DNA regions were cloned into the two-hybrid pKT25 and the ladS-D1 DNA region was cloned into pUT18C. All of the pKT25 construction as well as the empty vector were co-transformed in BTH101 cells with pUT18C vector containing or not ladS-D1 DNA regions and β-galactosidase activities were measured after 16 hours of growth. All experiments were carried out in at least triplicate, and error bars represent standard deviation. (B) The pBBRladS plasmid containing the ladS HK gene (dark bars) and the pBBRMCS4 corresponding empty cloning vector (light bars) were conjugated in the PAK, PAKΔhptA, PAKΔhptB, PAKΔhptC, PAKΔhptAΔhptBΔhptC, PAKΔrocS1, PAKΔrocS2, PAKΔPA4112, PAKΔPA4982 and PAKΔgacS strains. Activity of the rsmZ–lacZ (brick-red-colored) transcriptional chromosomal fusion was monitored after 6 hours of growth (OD600nm≈4) and corresponding β-galactosidase activities are expressed in Miller units and correspond to mean values (with error bars) obtained from three independent experiments. (TIF) [file pgen.1006032.s006.tif]
