# Supplementary material for: The Hybrid Histidine Kinase LadS Forms a Multicomponent Signal Transduction System with the GacS/GacA Two-Component System in Pseudomonas aeruginosa
Source: PLoS Genet. 2016 May 13;12(5):e1006032. doi: 10.1371/journal.pgen.1006032 (PMC4866733; doi:10.1371/journal.pgen.1006032)
Supplement: S1 Table — (DOCX) [file pgen.1006032.s007.docx]

| Names | Oligonucleotides (5’→3’) | |  |
| --- | --- | --- | --- |
| **Chromosomal fusions** |  | |  |
| PexoSUp | CCCAAGCTTTACGCTCTCCTCGTCGTTGG | |  |
| PexoSDn | CGGGATCCAATTCGACGGCGAAAGAGC | |  |
| PpelAUp | CCCAAGCTTAACTGAAGGCGGTGCGACCG | |  |
| PpelADn | CGGGATCCTGGGTCGAAGGATGTTACGG | |  |
| PrsmYUp | CGGAATTCAAGGCTCGCGATGATGAGG | |  |
| PrsmYDn | GGGGTACCTTTGGCGCTTCCTGCGCAATGTCC | |  |
| PrsmZUp | CCGGAATTCCCTTAGACCCACTGAAGACC | |  |
| PrsmZDn | GGGGTACCATCCTTCGGGGTTGCGTGTTCC | |  |
|  |  | |  |
| **Proteins production** |  | |  |
| LadSD1Up  LadSD1Dn  GacSH1D1Up  GacSH1D1Dn  GacSH1Up  GacSH1Dn  GacSD1Up  GacSD1Dn  LadSH1D1Up | TACTTCCAATCAATGGCCGGCGGGCAGGCGGTGCGGCGT  TATCCACCTTTACTGTTATTAGGCGGACTTGGTGACGATCGGCTGG  TACTTCCAATCAATGGGCAGCAACGAGCTG  TATCCACCTTTACTGTTATTACAGCGACTGGCCCAGGCTCAGTC  TACTTCCAATCAATGGGCAGCAACGAGCTG  TATCCACCTTTACTGTTATTAACTTTTCGGCAGACTCAG  TACTTCCAATCAATGGCCATGGTTTCCGGACGGCC  TATCCACCTTTACTGTTATTACAGCGACTGGCCCAGGCTCAGTC  GGAATTCACTTTAAGAAGGAGATATACCATGCATCCTCCAGGAGTCCAGCC | |  |
| LadSH1D1Dn | CGGGATCCTCAATGGTGATGGTGATGGTGGGCGGACTTGGTGACGATCGG | |  |
| GacSH2Up | GGGATCCACTTTAAGGAGATATACCATGCAGTCGCTGGCCAGCATGAGCCGTG | |  |
| GacSH2Dn  HptAUp  HptADn | CGAAGCTTTCAATGGTGATGGTGATGGTGGAGTTCGCTGGAGTCGAGGC  GGAATTCACTTTAAGAAGGAGATATACCATGAAAGAGCTTGGTTCGGAATCGC  AAGGATCCTCAATGGTGATGGTGATGGTGGAGCTTTGCCAATTCGGTTGAGATACTG | |  |
| **Pull Down experiments**  PDStrepLadSH1Up  PDFLAGLadSH1Up  PDLadSH1Dn  PDStrepGacSH2Up  PDFLAGGacSH2Up  PDGacSH1Dn  PDStrepRetSH1Up  PDFLAGRetSH1Up  PDRetSH1Dn | GCTCTAGAGAGCGCAGGAGCTGATGGCAAGCTGGAGCCACCCGCAGTTCGAAAAA  GGTGCAATCCTCCAGGAGTCCAGCC  GCTCTAGAGAGCGCAGGAGCTGATGGACTATAAAGACGACGACGACAAAATCCTC  CAGGAGTCCAGCC  CCGAGCTCTTACACCACCAGCACCGTGCACTCC  AACTGCAGGAGCGCAGGAGCTGATGGCAAGCTGGAGCCACCCGCAGTTCGAAAAA  GGTGCACTGCGCATGAGCCGCGCGATCAACGCGCCG  AACTGCAGGAGCGCAGGAGCTGATGGACTATAAAGACGACGACGACAAACTGGAA  CTGATCAGCCAGGGCGTCGC  GCTCTAGATTAGCCCGGCTCCTCGTTGTCGTCG  GCTCTAGAGAGCGCAGGAGCTGATGGCAAGCTGGAGCCACCCGCAGTTCGAAAAA  GGTGCAATCCAGCAGCTCAACCTGCAACAGCGC  GCTCTAGAGAGCGCAGGAGCTGATGGACTATAAAGACGACGACGACAAAATCCAG  CAGCTCAACCTGCAACAGCGC  CCGAGCTCTTAGAGGTCGGCGGTGGGGTTCTCCAGCTG | | |
|  | |  |  |
| **Quick exchange mutations** | |  |  |
| LadSQ1D1Up | | CTCGCCACCGTCACCCAAGAACTGCGCACCCCGATGAGG |  |
| LadSQ1D1Dn | | GTGCGCAGTTCTTGGGTGACGGTGGCGAGGAACTCGTC |  |
| LadSH1A1Up | | ACGGCGTACTGCTCGCGTGCCAGATGCCGGTGATGGACG |  |
| LadSH1A1Dn | | GCATCTGGCAGTCGAGCAGTACGCCGTCCGGCCGCTC |  |
| GacSQ2Up | | TTGCTCGAGAGGGTCCAACGGCTGCATGGCGCCACCC |  |
| GacSQ2Dn | | GGCGCCATGCAGCCGTTGGACCCTCTCGAGCAAAGCG |  |
| **Punctual chromosomal mutations**  UpULadS_D→A_  UpDLadS_D→A_  DnULadS_D→A_  DnDLadS_D→A_  UpULadS_H→Q_ UpDLadS_H→Q_  DnULadS_H→Q_ DnDLadS_H→Q_  UpUGacSH1_H→Q_  UpDGacSH1_H→Q_  DnUGacSH1_H→Q_  DnDGacSH1_H→Q_  UpUGacSH2_H→Q_  UpDGacSH2_H→Q_  DnUGacSH2_H→Q_  DnDGacSH2_H→Q_ | | CGGGATCCACAACGCGATCAAGTTCGCC  TCACCGGCATCTGGCAGGCGAGCAGTACGC  CTGCTCGCCTGCCAGATGCCGGTGATGGACGG  GGACTAGTAAGTGGCGGTACAGGCTGCC  CGGGATCCTACCAGGTCTCGGTGAACGG  GTGCGCAGTTCTTGGGTGACGGTGGCGAGGAACTCGTC  CTCGCCACCGTCACCCAAGAACTGCGCACCCCGATGAGC  GGACTAGTGGACGAAACGCTGGTAGAGG  GATTACGCGTTAACCCGGGCCCCCAGCCATTTGAGCATGTCC  GCGGATCTCTTGGCTCATGTTGG  CCAACATGAGCCAAGAGATCCGC  GGACTATAGACTATACTAGTGCTGAAGGCCTTGAACAAGG  GGGGATCCGTCACCGCGGTGGACAGCGG  GCCATGCAGCCGTTGGACCCTCTCGAGCAAAGCG  CTCGAGAGGGTCCAACGGCTGCATGGCGCCACCC  CCCACTAGTCCGGCAGGCCGAGCGCTTCG |  |
| **Gene Deletion** | |  |  |
| UpUPA4116 | | AAGGATCCGCTTCCTGCTGGCCGCGAT |  |
| UpDPA4116 | | TCAGCTCGCCGACTTGTACGTTCAC |  |
| DnUPA4116 | | AACAGAGCAAGTAGTCGGCGAGCTGA |  |
| DnDPA4116 | | CCCACTAGTGCGCCTGGAGGCGCCCGG |  |
| UpUPA4982 | | AAGGATCCCAACGACGAGATCGATCGC |  |
| UpDPA4982 | | TCAACCGGCCGCCGCAGAGCGCCAGCCA |  |
| DnUPA4982 | | AGCGGCCGGTTGAATGGCTGCTGG |  |
| DnDPA4982 | | CCCACTAGTGTGGAGGCCCAGCTCATCC |  |
| UpULadSD1 | | CGCGGATCCATGGTCAACGACATCCTCGC |  |
| UpDLadSD1 | | AAAGAATTCGTTGATGGCGTTGTCCTCCA |  |
| DnULadSD1 | | AAAGAATTCAAGTCCGCCTGAAGCCGTTC |  |
| DnDLadSD1 | | GGACTAGTGGTCACTTCGTGGGTTTCGG |  |
|  | |  |  |
| **Two-Hybrid** | |  |  |
| UDHLadSH1 | | GCTCTAGAGATCCTCCAGGAGTCCAGCC |  |
| DDHLadSH1 | | CCGAGCCCTCCACCACCAG |  |
| UDHHptA | | AAAAGCTGCAGGAGTCTGTATGAAAGAGCTTGGTTCGGAA |  |
| DDHHptA | | CGGGGTACCATTCTGAAACCTCTATCTGGTCCGATCTCTA |  |
| UDHHptB | | GCTCTAGAGCGAATGTCCGCGCCGCATCTCGATGATCGTG |  |
| DDHHptB | | GGGCCGGTACCTTGTCGCCGGAAAGGACGAAAACCTCAGC |  |
| UDHHptC | | GCTCTAGAGTCGCCCGCCGCCACGAGCTCATCTGAGGA |  |
| DDHHptC | | CGGGGTACCAGATCCTGGACGAAGGCCCGCAACGATATGA |  |
| UDHPA4112H2 | | GCTCTAGAGCAGATGCCGGATATCGATGGTC |  |
| DDHPA4112H2 | | CGGGGTACCATCAGCTCGCCGACTCCGCC |  |
| UDHPA4982H2 | | GCTCTAGAGGGACGCCGCGCTGGACC |  |
| DDHPA4982H2 | | CGGGGTACCATCAACCGGCCGCTGAG |  |
| UDHLadSD1 | | AAACTGCAGCTTCCTCCTGCGCCTGCAACTGACC |  |
| DDHLadSD1 | | GCATCGATGCGCGGAACGGCTTCAGGCGGACTTGG |  |
|  | |  |  |
| **RT-qPCR** | |  |  |
| rsmYUp | | AGGACATTGCGCAGGAAG |  |
| rsmYDn | | GGGGTTTTGCAGACCTCTC |  |
| rsmZUp | | CGTACAGGGAACACGCAAC |  |
| rsmZDn | | GTATTACCCCGCCCACTCTT |  |
| pelAUp | | CAGGTGCTGGAGGACTTCAT |  |
| pelADn | | AGAACGGATGGCTGAAGGTA |  |
| exoSUp | | CTCTACACCGGCATTCACTA |  |
| exoSDn | | CTTCACTACCTGTTCAGCCT |  |
| gacSH2RT-PCRUp | | TTGCTCGAGAGGGTCAC |  |
| gacSH2RT-PCRDn  vgrG1bUp  vgrG1bDn | | TCAGAGTTCGCTGGAGTCG  CCATTTCTACGACTGGCACA  GGGTAGTCGTACAAGCG |  |
| uvrDUp | | CACGCCTCGCCCTACAGCA |  |
| uvrDDn | | GGATCTGGAAGTTCTCGCTCAGC |  |
| ladSUp  ladSDn | | CAACGCGATCAAGTTCACCG  TGGACGAAACGCTGGTAGAG |  |
| 16SUp | | CAGCTCGTGTCGTGAGATGT |  |
| 16SDn | | GATCCGGACTACGATCGGTT |  |
